# The Impact of Single Amino Acids on Growth and Volatile Aroma Production by *Saccharomyces cerevisiae* Strains

**DOI:** 10.3389/fmicb.2017.02554

**Published:** 2017-12-19

**Authors:** Samantha Fairbairn, Alexander McKinnon, Hannibal T. Musarurwa, António C. Ferreira, Florian F. Bauer

**Affiliations:** ^1^Department of Viticulture and Oenology, Institute for Wine Biotechnology, University of Stellenbosch, Stellenbosch, South Africa; ^2^Escola Superior de Biotecnologia, Universidad Católica Portuguesa, Porto, Portugal

**Keywords:** amino acids, nitrogen, wine yeast, growth kinetics, wine aroma

## Abstract

Nitrogen availability and utilization by *Saccharomyces cerevisiae* significantly influence fermentation kinetics and the production of volatile compounds important for wine aroma. Amino acids are the most important nitrogen source and have been classified based on how well they support growth. This study evaluated the effect of single amino acids on growth kinetics and major volatile production of two phenotypically different commercial wine yeast strains in synthetic grape must. Four growth parameters, lag phase, maximum growth rate, total biomass formation and time to complete fermentation were evaluated. In contrast with previous findings, in fermentative conditions, phenylalanine and valine supported growth well and asparagine supported it poorly. The four parameters showed good correlations for most amino acid treatments, with some notable exceptions. Single amino acid treatments resulted in the predictable production of aromatic compounds, with a linear correlation between amino acid concentration and the concentration of aromatic compounds that are directly derived from these amino acids. With the increased complexity of nitrogen sources, linear correlations were lost and aroma production became unpredictable. However, even in complex medium minor changes in amino acid concentration continued to directly impact the formation of aromatic compounds, suggesting that the relative concentration of individual amino acids remains a predictor of aromatic outputs, independently of the complexity of metabolic interactions between carbon and nitrogen metabolism and between amino acid degradation and utilization pathways.

## Introduction

During fermentation, yeast take up and metabolize amino acids and other nutrients to support growth and produce biomass. In the process, a range of volatile aroma compounds, including esters, higher alcohols, volatile fatty acids, carbonyls and sulfur compounds are produced. The production of many of these aroma impact compounds is directly dependent on the nitrogen sources that are present during fermentation (Dickinson et al., 1997; Hernández-Orte et al., 2002; Miller et al., 2007; Styger et al., 2011; Torrea et al., 2011; Gutiérrez et al., 2012; Silva Ferreira et al., 2014; Burin et al., 2015; Stribny et al., 2015). In part, for this reason, the metabolic fate and efficiency of amino acids have been studied extensively (Cooper, 1982; Monteiro and Bisson, 1992; Godard et al., 2007; Crépin et al., 2012, 2017; Ljungdahl and Daignan-Fornier, 2012). However, our understanding of the metabolic regulation of these pathways remains limited, as does our ability to predict the aromatic outcome of a fermentation based on the chemical composition of the grape must.

Grape must nitrogen concentration and composition are highly variable, affecting yeast metabolism and consequently wine aroma (Bell and Henschke, 2005). *Saccharomyces cerevisiae* differentially utilizes amino acids which have been classified according to their ability to support growth, measured as generation time, when present as the sole nitrogen source (Cooper, 1982; Ljungdahl and Daignan-Fornier, 2012). Alanine, arginine, asparagine, aspartate, glutamate, glutamine, and serine were classified as preferred nitrogen sources and all other nitrogen sources as either intermediate or non-preferred (Ljungdahl and Daignan-Fornier, 2012). These studies provide important foundational data sets for elucidating the impact of amino acid composition on yeast growth. However, such studies have mostly been carried out with laboratory strains and in conditions that are very different from those encountered during winemaking.

The impact of complex amino acid mixtures in real and synthetic grape must on the synthesis of aromatic metabolites has been the focus of extensive studies (Hernández-Orte et al., 2002, 2006; Garde-Cerdán and Ancín-Azpilicueta, 2008; Barbosa et al., 2009). Many of the findings link the formation of various aroma compounds such as fusel alcohols and fusel acids to the degradation of branched-chain and aromatic (BCAAs) amino acids, via the Ehrlich pathway (Ehrlich, 1907; Hazelwood et al., 2008). Nitrogen metabolism is highly complex, as its intermediates are also shared between other metabolic pathways, including carbon metabolism. Consequently, the different nitrogen treatments and fermentation media used by various studies have thus far yielded an incomplete understanding of the impact of amino acid composition on aroma compound formation in fermentative conditions. It remains impossible to predict the aromatic output of a yeast strain in a medium with a complex nitrogen mixture even when the fermentation medium is fully described. Therefore, it is important to establish a foundational data set to evaluate how individual amino acids affect yeast growth, and aroma formation under fermentative conditions, and to establish to what degree such data can be extrapolated to predict the production of aromatic compounds when more complex nitrogen sources are used. To our knowledge, this is the first study to provide a comprehensive analysis of the growth kinetics of commercial wine yeast strains in response to single amino acids, as well as assessing the relationships between amino acid concentration and the formation of volatile compounds in simple and more complex matrices.

## Materials and methods

### Yeast strains

Two industrial *S. cerevisiae* strains VIN13 (Anchor Yeast, Cape Town, South Africa) and BM45 (Lallemand Inc., Montreal, Canada) were used, due to reported differences in fermentation kinetics and the resultant aroma profiles. The pre-culture procedure was as follows: A single yeast colony was inoculated into 100 mL YPD and incubated overnight with agitation at 30°C. Cultures were centrifuged and washed with sterile distilled water and used to inoculate 100 mL YPD broth at an OD_600nm_ of 0.1 (approximately 10^6^ CFU/mL) and again incubated overnight at 30°C. Cells were then centrifuged, washed, and resuspended in sterile distilled water. The fermentation media was then inoculated at an optical density of 0.1 at 600 nm.

### Media

Two fermentation media were used for this study; Yeast Nitrogen Base (YNB) without amino acids and ammonium (Difco™ Laboratories) and synthetic grape must (SGM) (Henschke and Jiranek, 1993).

Fermentations (100 mL) were conducted in triplicate at 30°C. The medium contained 10% m/v glucose, to ensure that alcoholic fermentation would go to completion, and the initial pH was adjusted to 3.8 with KOH or HCl. The source of ammonium (${\text{NH}}_{4}^{+}$) utilized was ammonium sulfate unless indicated otherwise.

The YNB media contained one of 19 amino acids or ${\text{NH}}_{4}^{+}$ as the sole source of yeast assimilable nitrogen (YAN) at a concentration of 10.71 mmol N/L.

The SGM contained acids (3.0 g/L potassium hydrogen tartaric acid, 2.5 g/L L-malic acid, and 0.2 g/L citric acid), salts (1.14 g/L K_2_HPO_4_, 1.23 g/L MgSO_4_.7H_2_O, and 0.44 g/L CaCl_2_.2H_2_O), trace elements (200 μg/L MnCl_2_.4H_2_O, 135 μg/L ZnCl_2_, 30 μg/L FeCl_2_, 15 μg/L CuCl_2_, 5 μg/L H_3_BO_3_, 30 μg/L Co[NO_3_]_2_.6H_2_O, 25 μg/L NaMoO_4_.2H_2_O, and 10 μg/L KIO_3_), vitamins (100 mg/L myo-inositol, 2 mg/L pridoxine.HCl, 2 mg/L nicotinic acid, 1 mg/L Ca-pantothenate, 0.5 mg/L thiamin.HCl, 0.2 mg/L para-aminobenzoic acid, 0.2 mg/L riboflavin, 0.125 mg/L biotin, and 0.2 mg/L folic acid), anaerobic factors (10 mg/L ergosterol and 0.5 mL/L Tween 80), and nitrogen sources as specified. The SGM contained a total YAN of 21.43 mmol N/L containing a single BCAA; phenylalanine, isoleucine, leucine, or valine at concentrations of 7.14, 14.28, and 21.43 mmol N/L (Table 1). The BCAAs treatments were supplemented, when required, with ${\text{NH}}_{4}^{+}$ for a total nitrogen content of 21.43 mmol N/L. An additional set of valine treatments were supplemented with alanine, instead of ammonium to investigate the effect of varying ammonium additions. Additional control treatments contained only ${\text{NH}}_{4}^{+}$ or alanine as the sole nitrogen source at a concentration of 21.43 mmol N/L.

**Table 1:** Amino acid treatments containing different concentrations of a branched-chain or aromatic amino acid and supplemented with either ${\text{NH}}_{4}^{+}$ or alanine to obtain a total YAN of 21.43 mmol N/L.

**Nitrogen treatment**	**BCAA concentration (mmol N/L)**	**${\text{NH}}_{4}^{+}$ (mmol N/L)**	**Ala (mmol N/L)**
Leu 21.43	21.43	0	0
Leu 14.28	14.28	7.14	0
Leu 7.14	7.14	14.28	0
Ile 21.43	21.43	0	0
Ile 14.28	14.28	7.14	0
Ile 7.14	7.14	14.28	0
Val 21.43	21.43	0	0
Val 14.28	14.28	7.14	0
Val 7.14	7.14	14.28	0
Ala 14.28 + Val 7.14	7.14	0	14.28
Ala 21.43 + Val 0	0	0	21.43
Ala 7.14 + Val 14.28	14.28	0	7.14
Phe 21.43	21.43	0	0
Phe 14.28	14.28	7.14	0
Phe 7.14	7.14	14.28	0
${\text{NH}}_{4}^{+}$	21.43	21.43	0

The third set of fermentations were conducted in SGM, containing 20% sugars and 200 mg N/L nitrogen. The nitrogen composition consisted of ammonium chloride (3.57 mg N/L), isoleucine (2.41 mg N/L), leucine (2.41 mg N/L), phenylalanine (2.41 mg N/L), tyrosine (2.41 mg N/L), and valine (2.41 mg N/L).

A final set of fermentations was conducted in SGM, as described above, however in this instance more complex mixtures of nitrogen (Table 2) were used. A YAN content of 14.3 mmol N/L was used, of which 3.57 mmol N/L was provided by ammonium chloride, the remainder was made up of all the amino acids, where each amino acid provided equal amounts of fermentable nitrogen. Furthermore, a single BCAA (leucine, isoleucine, valine, phenylalanine, tyrosine, and tryptophan) was either present at the same concentration as the other amino acids, absent (0) or present at twice (2) the concentration of the other amino acids. Fermentations were conducted in triplicate at 20°C without agitation.

**Table 2:** Fermentations contained 14.3 mmol N/L of YAN, of which 3.57 mmol N/L^−1^ was provided by ammonium chloride, the remainder was made up of all the amino acids listed, where each one provided equal amounts of fermentable nitrogen except for leucine, isoleucine, valine, phenylalanine, tyrosine, and tryptophan which were also either absent (0) or present at twice (2) the concentration of the other amino acids.

**Nitrogen sources (mmol N/L)**	**All amino acids**	**0 Ile**	**2 Ile**	**0 Leu**	**2 Leu**	**0 Val**	**2 Val**	**0 Trp**	**2 Trp**	**0 Tyr**	**2 Tyr**	**0 Phe**	**2 Phe**	**NH4^+^**
Alanine	0.59	0.63	0.56	0.63	0.56	0.63	0.56	0.63	0.56	0.63	0.56	0.63	0.56	0.00
Arginine	0.59	0.63	0.56	0.63	0.56	0.63	0.56	0.63	0.56	0.63	0.56	0.63	0.56	0.00
Aspartic acid	0.59	0.63	0.56	0.63	0.56	0.63	0.56	0.63	0.56	0.63	0.56	0.63	0.56	0.00
Cysteine	0.59	0.63	0.56	0.63	0.56	0.63	0.56	0.63	0.56	0.63	0.56	0.63	0.56	0.00
Glutamic acid	0.59	0.63	0.56	0.63	0.56	0.63	0.56	0.63	0.56	0.63	0.56	0.63	0.56	0.00
Glutamine	0.59	0.63	0.56	0.63	0.56	0.63	0.56	0.63	0.56	0.63	0.56	0.63	0.56	0.00
Glycine	0.59	0.63	0.56	0.63	0.56	0.63	0.56	0.63	0.56	0.63	0.56	0.63	0.56	0.00
Histidine	0.59	0.63	0.56	0.63	0.56	0.63	0.56	0.63	0.56	0.63	0.56	0.63	0.56	0.00
Lysine	0.59	0.63	0.56	0.63	0.56	0.63	0.56	0.63	0.56	0.63	0.56	0.63	0.56	0.00
Methionine	0.59	0.63	0.56	0.63	0.56	0.63	0.56	0.63	0.56	0.63	0.56	0.63	0.56	0.00
Proline	0.59	0.63	0.56	0.63	0.56	0.63	0.56	0.63	0.56	0.63	0.56	0.63	0.56	0.00
Serine	0.59	0.63	0.56	0.63	0.56	0.63	0.56	0.63	0.56	0.63	0.56	0.63	0.56	0.00
Threonine	0.59	0.63	0.56	0.63	0.56	0.63	0.56	0.63	0.56	0.63	0.56	0.63	0.56	0.00
Isoleucine	0.59	0.00	1.13	0.63	0.56	0.63	0.56	0.63	0.56	0.63	0.56	0.63	0.56	0.00
Leucine	0.59	0.63	0.56	0.00	1.13	0.63	0.56	0.63	0.56	0.63	0.56	0.63	0.56	0.00
Valine	0.59	0.63	0.56	0.63	0.56	0.00	1.13	0.63	0.56	0.63	0.56	0.63	0.56	0.00
Tryptophan	0.59	0.63	0.56	0.63	0.56	0.63	0.56	0.00	1.13	0.63	0.56	0.63	0.56	0.00
Tyrosine	0.59	0.63	0.56	0.63	0.56	0.63	0.56	0.63	0.56	0.00	1.13	0.63	0.56	0.00
Phenylalanine	0.59	0.63	0.56	0.63	0.56	0.63	0.56	0.63	0.56	0.63	0.56	0.00	1.13	0.00
Ammonium	3.57	3.57	3.57	3.57	3.57	3.57	3.57	3.57	3.57	3.57	3.57	3.57	3.57	14.29
Total YAN	14.3	14.3	14.3	14.3	14.3	14.3	14.3	14.3	14.3	14.3	14.3	14.3	14.3	14.3

### Growth parameters

#### Biomass

Optical density, measured at a wavelength of 600 nm (OD_600nm_), was used as an indication of biomass accumulation during fermentation and samples were taken every 2–4 h for the first 20 h followed by every 6–24 h until weight loss ceased. Two millilitres of the medium was aseptically collected from the fermentation vessel, centrifuged at 5,000 rpm for 5 min, and the supernatant was removed. Pellets were resuspended in distilled water, centrifuged, and washed again. Samples were dried for 48 h at 100°C and weighed. To obtain a calibration curve for the biomass, the dry weight was plotted against the OD reading at each of these sampling points.

#### Exponential growth rate and lag phase

The average exponential growth rate was determined by performing a semi-log transformation of the growth curve of each single amino acid treatment. From this transformed growth curve, the linear section was isolated, a trendline was added and its gradient represented the exponential growth rate. Since the lag phase ends where the exponential growth phase begins, the average lag phase was taken to be the period between the time of inoculation and the onset of exponential growth.

#### Time to complete fermentation

Fermentation vessels were weighed every 24 h until no further weight loss was detected.

### Gas chromatography with a flame ionization detector (GC-FID)

At the end of alcoholic fermentation, samples underwent a liquid-liquid extraction as described by Louw et al. (2009) for analysis by gas chromatography. A 5 mL sample of media, 100 μl of internal standard (4-methyl-2-pentanol), and 1 mL of solvent (diethyl ether) were combined and then placed in an ultrasonic bath for 5 min to facilitate extraction. The mixture was then centrifuged for 3 min at 4000 rpm after which Na_2_SO_4_ was added to remove any water from the non-polar layer and the sample was again centrifuged for another 3 min at 4000 rpm. A Hewlett Packard 6890 Plus GC-FID instrument (Agilent, Little Falls, Wilmington, USA) with a split/splitless injector was used for major volatiles quantification. The split flow rate was set at 49.4 ml/min and the split ratio was set to 15:1 at a temperature of 200°C. The separation of compounds was done using a J and B DBFFAP capillary GC column (Agilent, Little Falls, Wilmington, USA) with the dimensions of 60 m × 0.32 mm and a 0.5 μl coating film thickness with the flow rate of the hydrogen carrier gas set at 3.3 ml/min. Once the FID oven temperature reached the temperature of 240°C, 3 μl of extracted sample was injected into the gas chromatograph at an initial temperature of 33°C and held for 8 min; the temperature was then increased by 21°C/min to 130°C and then held for 17 min; increased by 12°C/min to 170°C and held for 5 min; increased by 21°C/min to 240°C and held for 2.5 min. A post run step at the end of each sample was carried out at 240°C for 5 min. Each sample was injected in duplicate. The column was cleaned with an injection of hexane after every 20 samples. Manual data collection and peak integration were done using the HP ChemStation software [Rev. B01.03 (204)].

### Prediction of aroma compound production

To assess the functional relationship between the final BCAAs concentration and their associated volatile metabolites accumulation, a linear relationship was assumed, and regression analysis was carried out to determine the association.

The assumed relationship can be summarized by the following equation:

$$\begin{array}{l} Y=a+bX \end{array}$$

Where *Y* = metabolite concentration; *b* = gradient of the slope; *X* = concentration of amino acid; *a* = metabolite concentration when no amino acid is added.

Different concentrations of BCAAs were plotted against their corresponding aroma compounds and the strength of the regression model represents the predictability of aroma compound production as a function of initial concentration.

### Statistical analysis

Heatmaps of autoscaled GC-FID data was constructed using the package Complex Heatmap in R studio, where applicable, rows and columns were clustered using Ward.D and the Euclidean distance metric. Similarly, cluster analysis was applied to the four yeast growth parameters measured.

## Results

### Effect of single amino acids on growth kinetics

**Figure 1:**
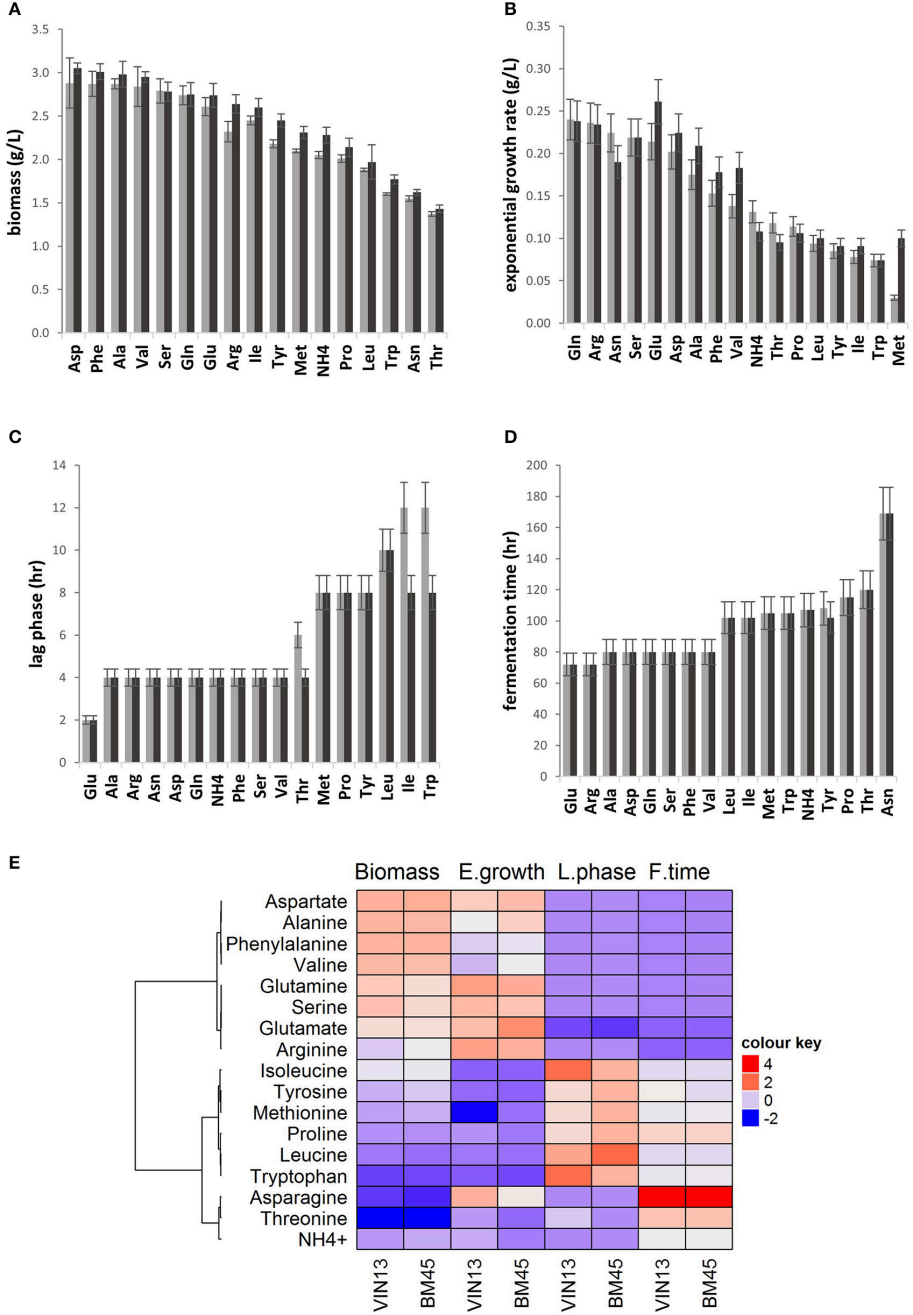
A comparison of biomass accumulation **(A)**, exponential growth rate **(B)** estimate of lag phase **(C)**, and fermentation duration **(D)** of VIN13 (light gray) and BM45 (dark gray) cultured in YNB containing a single amino acid or ammonium. Error bars denote the standard deviation of triplicate fermentations. The heatmap **(E)** summarizes all four growth parameters measured, where high values are colored red and low blue and the color intensity represents variation in the levels across the color scale.

Individual amino acids were evaluated for their ability to support the growth of VIN13 and BM45 in YNB. Differences in growth kinetics were observed due to both the amino acid treatment and, to a lesser extent, the yeast strain. Four growth parameters were assessed: the duration of the lag phase, exponential growth rate, total biomass formation and time to complete fermentation (Figures 1A–D). In most cases, and as expected, the four parameters showed a strong correlation, with short lag phase correlating with rapid exponential growth and high total biomass formation, as well as a shorter time of fermentation. In most cases; both strains also behaved similarly, but some significant exceptions were observed.

Glutamate, a preferred nitrogen source, resulted in the shortest lag phase (Figure 1C) in both strains, as well as a rapid exponential growth rate (Figure 1B), large biomass formation (Figure 1A) and short fermentation duration (Figure 1D). Glutamine, arginine, alanine, serine, aspartate, phenylalanine, and valine also generally supported similar patterns (Figure 1E). However, some significant differences were observed between the two strains, as glutamate and asparagine led to very different exponential growth rates for BM45 while remaining very similar in the case of VIN13 (Figure 1B).

For some amino acids, the general correlation between the four parameters was not maintained. This is illustrated by isoleucine, which yielded biomass comparable to the preferred amino acids such as arginine but resulted in a low exponential growth rate and long duration of fermentation (Figure 1E).

Tryptophan, threonine, and asparagine treatments resulted in the lowest biomass production levels for both yeast strains (Figure 1A). The yeast strain significantly impacted biomass production as seen in the arginine, isoleucine, leucine, threonine, tyrosine, methionine, and ${\text{NH}}_{4}^{+}$ treatments with VIN13 giving rise to lower final biomass (Figure 1A).

As summarized in the heatmap, isoleucine, tyrosine, methionine, proline, leucine, and tryptophan displayed a comparatively weaker performance in all four growth parameters evaluated when compared to the preferred amino acids seen in the first cluster.

Asparagine, threonine, and ammonia are in the third cluster, as they displayed short lag phases, despite having low levels of biomass production. Ammonia is considered to be a preferred nitrogen source and therefore its poor performance was further investigated, and this deviant behavior was ascribed to the buffering capacity of the YNB media used (data not shown).

Yeast strains also responded differently to certain nitrogen treatments, as shown by the longer lag phases observed for VIN13 when supplemented with only threonine, isoleucine, and tryptophan (Figure 1C). The highest growth rate was observed with the BM45 strain in the glutamate treatment (Figure 1B). Lower growth rates were observed in both strains for ${\text{NH}}_{4}^{+}$, threonine, proline, methionine, leucine, tyrosine, isoleucine, and tryptophan treatments.

### Effect of single amino acids on aroma production

**Figure 2:**
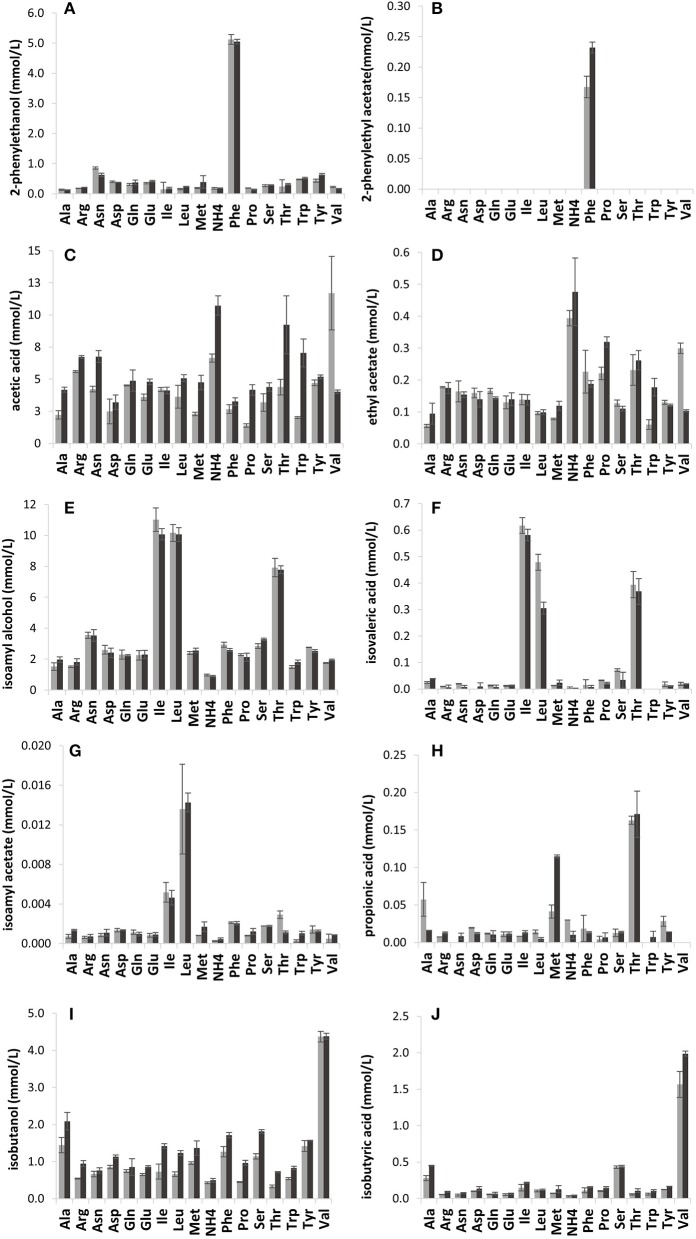
The impact of single amino acids in YNB on the production of volatile compounds by VIN13 (light gray) and BM45 (dark gray) on the production of volatile compounds (mmol/L) such as 2-phenylethanol **(A)**, 2-phenylethyl acetate **(B)**, acetic acid **(C)**, ethyl acetate **(D)**, isoamyl alcohol **(E)**, isovaleric acid **(F)**, isoamyl acetate **(G)**, propionic acid **(H)**, isobutanol **(I)**, and isobutyric acid **(J)**. Error bars denote the standard deviation of triplicate fermentations.

As anticipated, the volatile compound composition was strongly influenced by the amino acid (21.43 mmol N/L) provided (Figure 2). The branched-chain and aromatic amino acid treatments yielded high concentrations of fusel alcohols and fusel acids mostly in line with the known metabolic pathways. Valine treatment resulted in a high production of isobutanol, (4.4 mmol/L) and isobutyric acid, where VIN13 produced 1.57 mmol/L and BM45 1.99 mmol/L (Figures 2I,J). The leucine and isoleucine treatments were linked to isoamyl alcohol, isovaleric acid and isoamyl acetate (Figures 2E–G) production. The products of isoleucine (amyl alcohol and 2-methylbutanoic acid) and leucine (isoamyl alcohol and isovaleric acid) catabolism have similar structures and consequently also similar retention times. The misidentification of metabolites is most likely the reason why the profile so closely resembles that of leucine. Phenylalanine metabolism led to the production of more than 5 mmol/L of 2-phenylethanol, by both strains, and 0.17 mmol/L of 2-phenylethyl acetate by VIN13 and 0.23 mmol/L by BM45 (Figures 2A,B). Threonine catabolism resulted in the production of propanol but also resulted in the highest levels of butanol and propionic acid, moderate levels of isoamyl alcohol, isovaleric acid, acetic acid, and ethyl acetate (Figures 2C–F and H).

### Evaluating aroma production based on BCAAs concentration

**Figure 3:**
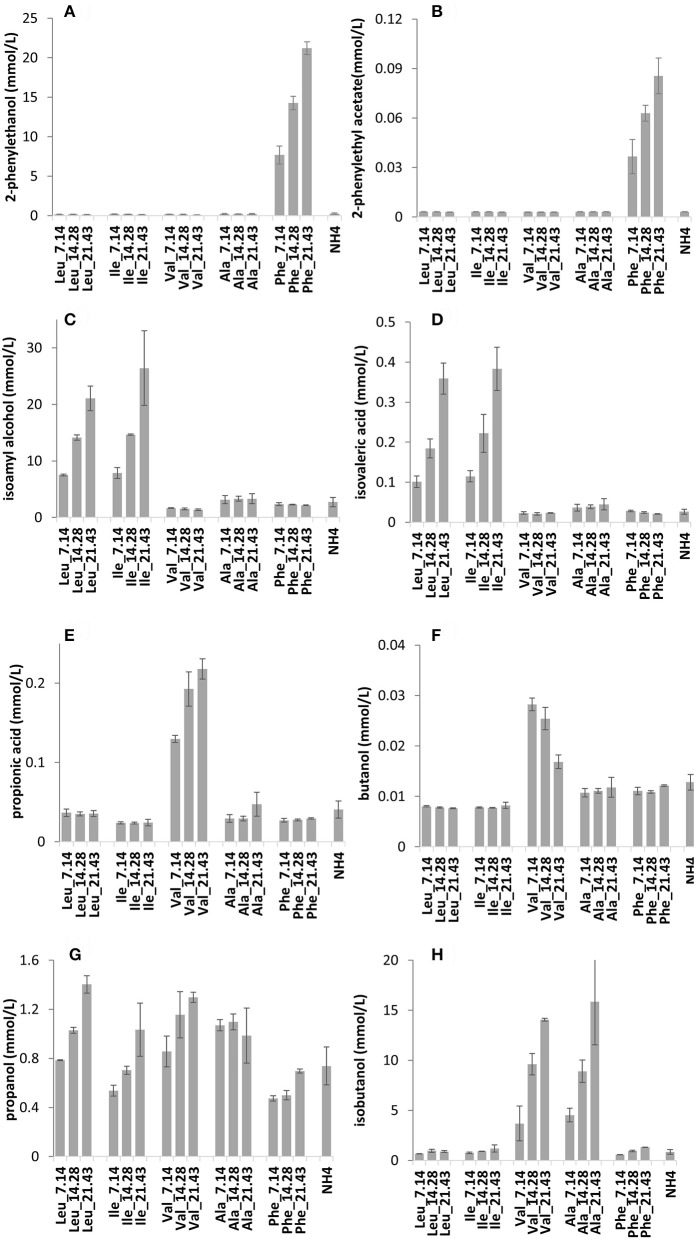
The impact of increasing concentrations of BCAAs in synthetic grape must on the production of volatile compounds by VIN13: 2-phenylethanol **(A)**, 2-phenylethyl acetate **(B)**, isoamyl alcohol **(C)**, isovaleric acid **(D)**, propionic acid **(E)**, butanol **(F)**, propanol **(G)**, and isobutanol **(H)**. Error bars denote the standard deviation of triplicate fermentations.

The effect of single BCAAs on aroma biosynthesis was further investigated using VIN13 in synthetic grape must. Three amino acid concentrations were applied while maintaining total available nitrogen through ammonium addition. To evaluate whether changing ammonium concentrations might impact the result, the same data were also produced using alanine instead of ammonium. Volatile acidity (acetic acid and ethyl acetate) displayed a proportional response to ammonium supplementation (Table S2), whereas propanol displayed an inverse relationship to supplementation (Figure 3G).

The data suggested that a linear correlation existed, consequently, data were used to develop a simple regression model to evaluate aroma compound production based on the initial amino acid concentration (Table 3).

**Table 3:** Linear regression analysis of the relationship between BCAAs and related volatile metabolite produced by VIN13 during fermentation.

**Amino acid**	**Aroma compound**	**R^2^**	**Regression equation**	***P-value***
Val	Isobutanol	0.9967	*Y* = −1.25 + 0.73X	0.052
	Isobutyric acid	0.9991	*Y* = −0.40 + 0.12X	0.019^*^
	Butyric acid	0.9999	*Y* = 0.002 − (2.06 × 10^−5^) X	0.008^*^
	Propionic acid	0.9414	*Y* = 0.09 + 0.006X	0.156
Phe	2-phenylethanol	0.9998	*Y* = 0.87 + 0.95X	0.009^*^
	2-phenylethyl acetate	0.9980	*Y* = 0.0034X + 0.0128	0.028^*^
	Decanoic acid	0.8878	*Y* = 0.01 − 0.0002X	0.217
	Valeric acid	0.9989	*Y* = 0.007 + 0.0007X	0.022^*^
Leu	Isoamyl alcohol	0.9999	*Y* = 0.69 + 0.95X	0.008^*^
	Isovaleric acid	0.9596	*Y* = −0.04 + 0.02X	0.129
	Isoamyl acetate	0.9927	*Y* = 0.02 + 0.002X	0.055
Ile	Isoamyl alcohol	0.9767	*Y* = 1.2988X − 2.2464	0.098
	Isovaleric acid	0.9869	*Y* = −0.03 + 0.02X	0.073
	Isoamyl acetate	0.2181	*Y* = 0.01 + 0.0003X	0.691

* *P-values with asterisk indicates a significant relationship between the amino acid concentration and the volatile compound produced (at 95% confidence level)*.

Irrespective of the addition of ammonia or alanine, the valine fermentations responded in a similar manner with respect to isobutanol and isobutyric acid, whereas butanol, and propionic acid levels were significantly impacted by ammonium levels (Figures 3E,F,H).

As shown in Figure 2 (Table S1), these SGM fermentations displayed a similar response to BCAA supplementation. A linear correlation between the concentration of an amino acid and the production of fusel alcohols and fusel acids was observed when the initial concentrations of BCAAs were varied individually (Figure 3, Table S2). Some volatile compounds related to valine (isobutyric acid and butyric acid), phenylalanine (2-phenylethanol and valeric acid) and leucine (isoamyl alcohol) could be predicted using the correlation analyses. Although other aroma compounds had high regression coefficients (R^2^), the slopes were insignificant (Table 3). Surprisingly, the total yield of aromatic compounds, primarily the higher alcohol, was identical in concentration to the total amount of leucine and phenylalanine (Table 4). This may suggest that the contribution of other metabolic pathways to these aromatic compounds is neg1egible in these conditions, or could be a fortuitous coincidence. In the case of valine, the measured catabolic products account for between 58 and 76% of the initial concentration, but we did not measure some of the major breakdown products in this case.

**Table 4:** The volatile aroma yield (%) based on the BCAA concentration and related volatile metabolite produced by VIN13 during fermentation.

				**Total yield (%)**
	**Phenylethyl alcohol**	**Phenylethyl acetate**		
Phe_7.14	107.58 ± 16.14	0.513 ± 0.15		108.10
Phe_14.28	100.00 ± 5.91	0.440 ± 0.03		100.44
Phe_21.43	98.98 ± 3.77	0.399 ± 0.05		99.38
	**Isoamyl alcohol**	**Isovaleric acid**	**Isoamyl acetate**	
Leu_7.14	105.08 ± 2.57	1.42 ± 0.2	0.424 ±0.04	106.92
Leu_14.28	98.93 ± 3.32	1.29 ± 0.17	0.320 ±0.04	100.54
Leu_21.43	98.20 ± 10.18	1.67 ± 0.18	0.267 ±0.03	100.14
Ile_7.14	110.00 ± 13.42	1.61 ± 0.2	0.130 ±0.009	111.74
Ile_14.28	102.57 ± 0.68	1.56 ± 0.33	0.127 ±0.023	104.25
Ile_21.43	117.05 ± 7.9	1.79 ± 0.25	0.063 ±0.02	118.90
	**Isobutanol**	**Isobutyric acid**		
Val_7.15	51.58 ± 2.09	7.03 ± 0.81		58.61
Val_14.28	67.23 ± 7.51	9.38 ± 1.02		76.61
Val_21.43	65.58 ± 8.1	10.59 ± 1.34		76.17

To evaluate the performance of these regression models, fermentations were conducted using more complex amino acid mixtures. These fermentations contained the BCAA's, isoleucine, leucine, phenylalanine, tyrosine, and valine, and the predicted concentrations of aromatic compounds were determined using the equations in Tables 3, 5. In this comparatively simple matrix, the actual concentrations determined differed from the predicted values, indicating that the predictive ability of the linear correlational analyses has already been lost.

**Table 5:** Comparison between the actual and predicted concentrations of volatile metabolites in response to mixtures of BCAAs produced by VIN13 during fermentation.

**Amino acid**	**Aroma compound**	**Measured concentration (mmol/L)**	**Predicted concentration (mmol/L)**
Leu and Ile	Isoamyl alcohol	4.7 ± 0.03	3.9
	Isovaleric acid	0.042 ± 0.001	0.018
	Isoamyl acetate	0.020 ± 0.001	0.032
Val	Isobutanol	1.7 ± 0.01	0.495
	Isobutyric acid	0.039 ± 0.000	−0.0981
	Propionic acid	0.023 ± 0.000	0.107
	Butyric acid	0.013 ± 0.000	0.002
Phe	2-phenyl ethanol	2.3 ± 0.03	3.1
	2-phenylethyl acetate	0.016 ± 0.001	0.021
	Valeric acid	0.007 ± 0.000	0.009
	Decanoic acid	0.014 ± 0.002	0.012

### Effect of changing the concentration of one amino acid in a complex mixture on aroma production

To evaluate the impact of relatively minor changes in amino acid composition, which more closely mimics grape must, fermentations were carried out using 19 amino acids, but varying only a single BCAA (leucine, isoleucine, valine, phenylalanine, tyrosine, and threonine) concentration. Three concentrations were used for each of these amino acids: Absent (0), same as all other amino acids and present at twice (2) the concentration of the other amino acids. The impact of this omission or twice the concentration of BCAA's on the production of aroma compounds was evaluated, in a more complex synthetic grape must (20% sugars). The amino acid treatments resulted in similar fermentation kinetics, with the ammonium treatment being comparatively slower, nonetheless, all fermentations proceeded to dryness (<2 g/L residual sugar, data not shown).

**Figure 4:**
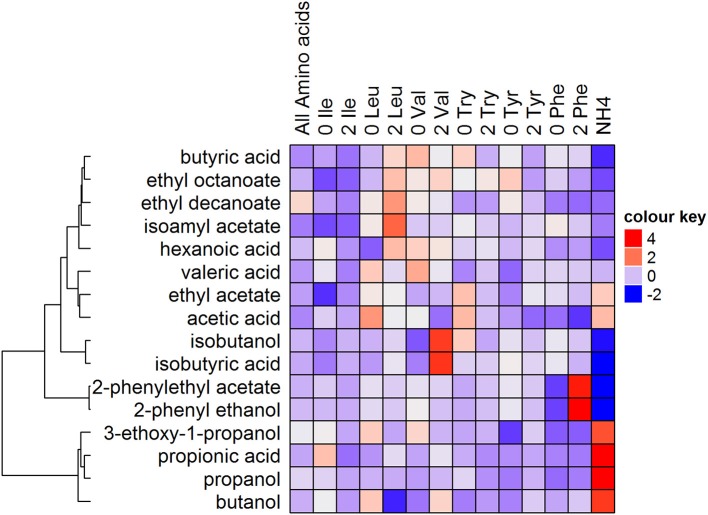
The impact of the absence (0) or twice the concentration of nitrogen (1.13 mmol N/L) (2) provided by BCCAs when all other amino acids provide the same amount of fermentable nitrogen on the production of volatile compounds (mmol/L). Fermentations, mediated by VIN13, were conducted in synthetic grape must.

The concentrations yielded differed from the predicted levels calculated, suggesting a loss of linear predictability. Nonetheless, the data indicates that these individual amino acid changes do indeed cause significant changes in the production compounds associated with their catabolism (Figure 4, Table S3). This is clearly illustrated by a responsiveness to leucine (isoamyl acetate), phenylalanine (2-phenyl ethanol and 2-phenylethyl acetate) and valine (isobutanol and isobutyric acid) supplementation, where the expected increases (2 treatments) or decreases (0 treatments) were observed. Relative to the all amino acid treatment (0.59 mmol N/L), the absence of phenylalanine resulted in a 56% decrease in the production of 2-phenyl ethanol, and a 46% reduction in 2-phenylethyl acetate. In contrast, twice the concentration of phenylalanine (1.13 mmol N/L) resulted in a 127% increase in the production of 2-phenyl ethanol and a 110% increase in 2-phenylethyl acetate. Similarly, the absence of valine resulted in a 24% decrease in isobutanol and 13% decrease in isobutyric acid, and twice the concentration of valine (1.13 mmol N/L) caused an 80% increase in isobutanol and isobutyric acid. The absence or twice the concentration of tryptophan and tyrosine only resulted in slight changes in the production of volatile fatty acids and ethyl esters. The ammonium treatment produced high levels of propanol, propionic acid, 3-ethoxy-1-propanol, and butanol, also seen in Figure 3, and very low levels of metabolites derived from phenylalanine and valine metabolism.

## Discussion

In a winemaking context, nitrogen has a dual role, promoting yeast growth to achieve completion of alcoholic fermentation, but also playing a central role in the production of volatile aromas. Previous studies used generation time to evaluate the efficiency of nitrogen sources to support growth (Cooper, 1982; Godard et al., 2007). This study increased the number of growth parameters evaluated, which allowed for a more rigorous and complete study of the efficiency of single amino acids in a wine-specific context. Since amino acids are provided as the sole source of nitrogen, no regulatory interference in their utilization occurs from the presence of other nitrogen sources (Godard et al., 2007; Ljungdahl and Daignan-Fornier, 2012). In general, the amino acid efficiency observed under fermentative conditions was in agreement with previous studies by Cooper (1982) and Godard et al. (2007). The preferred nitrogen sources, which best supported fermentation kinetics (alanine, arginine, aspartate, glutamine, glutamate, and serine), are also the amino acids to be readily incorporated into the cell's metabolic pathways. In contrast with previous findings, the phenylalanine and valine treatments resembled the preferred amino acids whereas asparagine was a poor nitrogen source based on its capacity to support yeast growth. These differences in amino acid classification could be attributed to different fermentation conditions, wine yeast strains used, as well as the additional parameters assessed.

Overall, biomass formation was generally correlated with the duration of the lag phase and the exponential growth rate correlated with the fermentation time. Exceptions to these correlations, due to strain differences, were found for both the biomass and lag phase (arginine, isoleucine, and threonine treatments) and exponential growth rate and fermentation time (valine and threonine treatments). Furthermore, asparagine was found to be an exception due to its irregular performance for all parameters evaluated. In a winemaking context, the inclusion of fermentation time and biomass provides a greater understanding of the effect of these individual amino acids on fermentation performance.

The type and quantity of volatile compounds produced are largely dependent on the amino acid, the yeast strain, and their interaction. Overall, similar responses to the nitrogen treatments were observed for most compounds, however, strains often differed in the magnitude of their responses to some of the amino acid treatments employed. In agreement with literature, the degradation of BCAAs into their corresponding fusel alcohols and fusel acids was observed to be positively linked (Hazelwood et al., 2008). Interestingly, valine was the only amino acid which resulted in less than a 100% conversion rate into its related volatile compounds. This suggests that the yeast redirected the alpha-keto acid (α-keto-isovalerate) toward a different metabolic pathway, possibly leucine biosynthesis. Whereas, irrespective of its concentration, leucine and phenylalanine resulted in the complete conversion of the amino acid to its volatile breakdown products, suggesting that all the amino acids were synthesized *de novo* (Crépin et al., 2017). Consequently, when single BCAAs are used together with ammonium as the only other source of nitrogen, aroma compound production shows a strong linear correlation between the amino acid concentration and the corresponding volatile compound (Figure 3). Subsequent regression analyses highlighted the potential of using amino acid concentration to predict the concentration of the associated volatile compound (Table 4), however, only isoamyl alcohol, isobutyric acid, butyric acid, 2-phenylethanol, and valeric acid could be reliably predicted using the regression models. Considering the interconnectedness of nitrogen metabolism, it is not surprising that when all BCAAs are used together, the linear relationship was lost resulting in poor predictability (Figure 4). Generally, amino acids are catabolised yielding glutamate, which is central to amino acid biosynthesis, and a carbon skeleton which can enter the TCA cycle, the Ehrlich pathway, or be used for the biosynthesis of other amino acids, as is the case with leucine and valine (Magasanik and Kaiser, 2002). Consequently, in the absence of leucine, one would assume a decrease in the levels of volatile compounds associated with valine catabolism, as the cell would presumably use the carbon skeleton to produce leucine instead of a fusel alcohol or acid (Figure 4). This is not the case, as explained by Crépin et al. (2017), in a recent study monitoring the fate of amino acids, which found that the α-keto acid precursors used for higher alcohol production were generally derived from sugar and not amino acid catabolism. The data shown here (Figure 4) illustrates that even comparatively minor changes in BCAA concentrations do still result in significant impacts on the production of the associated volatile compounds, even when provided in complex mixtures and the presence of all amino acids. Nonetheless, the work reported by Crépin et al. (2017) raises a concern that the application of nutrients during winemaking may not make a direct contribution to the volatile profile, but the data shown here clearly indicates that that is not the case as a degree of responsiveness to even minor changes in individual amino acid supplementation led to a direct impact on the production of associated aromatic compounds. Future work will explore prediction modeling of the impact of complex nitrogen composition on metabolic outputs.

This study provides a novel evaluation of the impact of single amino acids on several growth parameters, in addition to its effect on the production of volatile metabolites by two genetically different industrial *S. cerevisiae* strains, known to have differing fermentation kinetics and volatile aroma profiles (Rossouw and Bauer, 2009). The various correlations between amino acid concentration and volatile compounds ranged from linear in the simplest of all cases (single amino acid) to unpredictable in more complex media. However, and importantly, relatively minor changes in the concentrations of single amino acids still led to changes in the variation of the derived volatile compounds.

## Author contributions

SF and AM performed experiments, analyzed data, and wrote the manuscript; HM and AF wrote the manuscript; FB designed the experiments, contributed to data interpretation, supervised the study, and co-wrote the manuscript.

### Conflict of interest statement

The authors declare that the research was conducted in the absence of any commercial or financial relationships that could be construed as a potential conflict of interest.
