# Supplementary material for: The Impact of Single Amino Acids on Growth and Volatile Aroma Production by Saccharomyces cerevisiae Strains
Source: Front Microbiol. 2017 Dec 19;8:2554. doi: 10.3389/fmicb.2017.02554 (PMC5742263; doi:10.3389/fmicb.2017.02554)
Supplement: Supplementary file 1 [file Table1.docx]

Supplementary Material

**The impact of single amino acids on growth and volatile aroma production by *Saccharomyces cerevisiae* strains**

**Samantha Fairbairn^1^, Alexander McKinnon^1^, Hannibal T. Musarurwa^1^, António C Ferreira^1,2^ & Florian F Bauer^1*^**

^1^Institute for Wine Biotechnology, Department of Viticulture and Oenology, University of Stellenbosch, Stellenbosch South Africa

^2^Escola Superior de Biotecnologia, Universidad Católica Portuguesa, Rua Dr. António Bernardino de Almeida, 4200-072 Porto, Portugal

*** Correspondence:**

Florian F Bauer

[fb2@sun.ac.za](mailto:fb2@sun.ac.za)

**Keywords: amino acids, nitrogen, *Saccharomyces cerevisiae*, growth kinetics, wine aroma, predictive modelling**

# Table S1. The impact of single amino acids in YNB on the production of volatile compounds byVIN13 and BM45 (dark grey as determined by GC-FID. The data summarizes the average fermentations and standard deviation. Additionally, the letters denote significant differences (95%) between treatments using Fisher LSD.

|  | **Ethyl acetate** | | | | **Propanol** | | | | **Isobutanol** | | | |
| --- | --- | --- | --- | --- | --- | --- | --- | --- | --- | --- | --- | --- |
| **ALA_BM45** | 0.09 | ± | 0.03 | ^nop^ | 0.22 | ± | 0.03 | ^efghijk^ | 2.09 | ± | 0.24 | ^b^ |
| **ALA_VIN13** | 0.06 | ± | 0.01 | ^p^ | 0.30 | ± | 0.03 | ^def^ | 1.44 | ± | 0.21 | ^ef^ |
| **ARG_BM45** | 0.18 | ± | 0.02 | ^fghij^ | 0.15 | ± | 0.00 | ^ijkl^ | 0.94 | ± | 0.08 | ^jkl^ |
| **ARG_VIN13** | 0.18 | ± | 0.00 | ^fghi^ | 0.32 | ± | 0.00 | ^de^ | 0.54 | ± | 0.01 | ^opq^ |
| **ASN_BM45** | 0.15 | ± | 0.01 | ^ghijklm^ | 0.11 | ± | 0.02 | ^l^ | 0.76 | ± | 0.07 | ^lmn^ |
| **ASN_VIN13** | 0.16 | ± | 0.03 | ^ghijk^ | 0.18 | ± | 0.06 | ^ghijkl^ | 0.66 | ± | 0.08 | ^nop^ |
| **ASP_BM45** | 0.14 | ± | 0.02 | ^hijklmn^ | 0.14 | ± | 0.02 | ^jkl^ | 1.13 | ± | 0.05 | ^ij^ |
| **ASP_VIN13** | 0.16 | ± | 0.02 | ^ghijkl^ | 0.25 | ± | 0.02 | ^defgh^ | 0.85 | ± | 0.05 | ^klm^ |
| **GLN_BM45** | 0.14 | ± | 0.00 | ^hijklmn^ | 0.13 | ± | 0.00 | ^fghijkl^ | 0.85 | ± | 0.22 | ^klm^ |
| **GLN_VIN13** | 0.17 | ± | 0.01 | ^ghijk^ | 0.29 | ± | 0.02 | ^def^ | 0.74 | ± | 0.04 | ^mn^ |
| **ILE_BM45** | 0.14 | ± | 0.02 | ^hijklmn^ | 0.25 | ± | 0.01 | ^defghi^ | 1.42 | ± | 0.06 | ^efg^ |
| **ILE_VIN13** | 0.14 | ± | 0.02 | ^ghijklmn^ | 0.54 | ± | 0.08 | ^bc^ | 0.73 | ± | 0.21 | ^mno^ |
| **LEU_BM45** | 0.10 | ± | 0.01 | ^mnop^ | 0.28 | ± | 0.08 | ^defg^ | 1.23 | ± | 0.07 | ^hi^ |
| **LEU_VIN13** | 0.10 | ± | 0.01 | ^nop^ | 0.61 | ± | 0.04 | ^ab^ | 0.66 | ± | 0.06 | ^nop^ |
| **METH_BM45** | 0.12 | ± | 0.01 | ^klmno^ | 0.27 | ± | 0.02 | ^defg^ | 1.36 | ± | 0.20 | ^fgh^ |
| **METH_VIN13** | 0.08 | ± | 0.00 | ^op^ | 0.34 | ± | 0.02 | ^d^ | 0.96 | ± | 0.03 | ^jk^ |
| **NH4_BM45** | 0.48 | ± | 0.11 | ^a^ | 0.12 | ± | 0.02 | ^kl^ | 0.49 | ± | 0.05 | ^pqr^ |
| **NH4_VIN13** | 0.39 | ± | 0.02 | ^b^ | 0.29 | ± | 0.04 | ^defg^ | 0.43 | ± | 0.03 | ^qr^ |
| **PHE_BM45** | 0.19 | ± | 0.01 | ^fgh^ | 0.23 | ± | 0.01 | ^defghij^ | 1.71 | ± | 0.07 | ^cd^ |
| **PHE_VIN13** | 0.23 | ± | 0.07 | ^def^ | 0.34 | ± | 0.06 | ^d^ | 1.27 | ± | 0.14 | ^ghi^ |
| **PRO_BM45** | 0.32 | ± | 0.02 | ^c^ | 0.51 | ± | 0.04 | ^bc^ | 0.96 | ± | 0.07 | ^jk^ |
| **PRO_VIN13** | 0.22 | ± | 0.02 | ^efg^ | 0.66 | ± | 0.02 | ^a^ | 0.45 | ± | 0.01 | ^qr^ |
| **SER_BM45** | 0.11 | ± | 0.01 | ^klmnop^ | 0.15 | ± | 0.04 | ^hijkl^ | 1.81 | ± | 0.05 | ^c^ |
| **SER_VIN13** | 0.13 | ± | 0.01 | ^ijklmno^ | 0.30 | ± | 0.04 | ^def^ | 1.14 | ± | 0.07 | ^i^ |
| **THR_BM45** | 0.26 | ± | 0.03 | ^d^ | 0.47 | ± | 0.03 | ^c^ | 0.71 | ± | 0.01 | ^mno^ |
| **THR_VIN13** | 0.23 | ± | 0.05 | ^def^ | 0.46 | ± | 0.08 | ^c^ | 0.33 | ± | 0.04 | ^r^ |
| **TRP_BM45** | 0.18 | ± | 0.03 | ^fghi^ | 0.12 | ± | 0.01 | ^kl^ | 0.83 | ± | 0.05 | ^klmn^ |
| **TRP_VIN13** | 0.06 | ± | 0.01 | ^p^ | 0.15 | ± | 0.01 | ^hijkl^ | 0.54 | ± | 0.03 | ^pq^ |
| **TYR_BM45** | 0.12 | ± | 0.00 | ^jklmno^ | 0.29 | ± | 0.00 | ^def^ | 1.57 | ± | 0.01 | ^de^ |
| **TYR_VIN13** | 0.13 | ± | 0.01 | ^ijklmno^ | 0.47 | ± | 0.03 | ^c^ | 1.42 | ± | 0.15 | ^efgh^ |
| **VAL_BM45** | 0.10 | ± | 0.00 | ^lmnop^ | 0.59 | ± | 0.01 | ^ab^ | 4.38 | ± | 0.08 | ^a^ |
| **VAL_VIN13** | 0.30 | ± | 0.02 | ^de^ | 0.76 | ± | 0.03 | ^ab^ | 4.37 | ± | 0.14 | ^a^ |

**Table S1. continued**

|  | **Isoamyl acetate** | | | | **Butanol** | | | | **Isoamyl alcohol** | | | |
| --- | --- | --- | --- | --- | --- | --- | --- | --- | --- | --- | --- | --- |
| **ALA_BM45** | 0.00 | ± | 0.00 | ^defg^ | 0.00 | ± | 0.00 | ^ghij^ | 1.98 | ± | 0.17 | ^klmno^ |
| **ALA_VIN13** | 0.00 | ± | 0.00 | ^defg^ | 0.00 | ± | 0.00 | ^fghij^ | 1.53 | ± | 0.24 | ^op^ |
| **ARG_BM45** | 0.00 | ± | 0.00 | ^defg^ | 0.00 | ± | 0.00 | ^fghij^ | 1.82 | ± | 0.20 | ^lmnop^ |
| **ARG_VIN13** | 0.00 | ± | 0.00 | ^defg^ | 0.00 | ± | 0.00 | ^fghij^ | 1.50 | ± | 0.05 | ^op^ |
| **ASN_BM45** | 0.00 | ± | 0.00 | ^defg^ | 0.00 | ± | 0.00 | ^fghij^ | 3.53 | ± | 0.38 | ^d^ |
| **ASN_VIN13** | 0.00 | ± | 0.00 | ^defg^ | 0.00 | ± | 0.00 | ^fghij^ | 3.54 | ± | 0.20 | ^d^ |
| **ASP_BM45** | 0.00 | ± | 0.00 | ^cdefg^ | 0.00 | ± | 0.00 | ^hij^ | 2.42 | ± | 0.30 | ^ghijk^ |
| **ASP_VIN13** | 0.00 | ± | 0.00 | ^defg^ | 0.00 | ± | 0.00 | ^ghij^ | 2.61 | ± | 0.29 | ^fghi^ |
| **GLN_BM45** | 0.00 | ± | 0.00 | ^defg^ | 0.00 | ± | 0.00 | ^hij^ | 2.23 | ± | 0.05 | ^ijklmn^ |
| **GLN_VIN13** | 0.00 | ± | 0.00 | ^defg^ | 0.00 | ± | 0.00 | ^fghij^ | 2.30 | ± | 0.29 | ^hijkl^ |
| **ILE_BM45** | 0.00 | ± | 0.00 | ^b^ | 0.00 | ± | 0.00 | ^hij^ | 10.07 | ± | 0.37 | ^b^ |
| **ILE_VIN13** | 0.01 | ± | 0.00 | ^b^ | 0.0007 | ± | 0.0000 | ^efgh^ | 11.01 | ± | 0.76 | ^a^ |
| **LEU_BM45** | 0.01 | ± | 0.00 | ^a^ | 0.00 | ± | 0.00 | ^ij^ | 10.06 | ± | 0.45 | ^b^ |
| **LEU_VIN13** | 0.01 | ± | 0.00 | ^a^ | 0.00 | ± | 0.00 | ^fghij^ | 10.16 | ± | 0.54 | ^b^ |
| **METH_BM45** | 0.00 | ± | 0.00 | ^cdefg^ | 0.01 | ± | 0.00 | ^d^ | 2.56 | ± | 0.16 | ^fghij^ |
| **METH_VIN13** | 0.00 | ± | 0.00 | ^defg^ | 0.01 | ± | 0.00 | ^c^ | 2.40 | ± | 0.10 | ^ghijk^ |
| **NH4_BM45** | 0.00 | ± | 0.00 | ^fg^ | 0.00 | ± | 0.00 | ^fghij^ | 0.90 | ± | 0.06 | ^q^ |
| **NH4_VIN13** | 0.00 | ± | 0.00 | ^g^ | 0.00 | ± | 0.00 | ^efg^ | 0.98 | ± | 0.06 | ^q^ |
| **PHE_BM45** | 0.00 | ± | 0.00 | ^cde^ | 0.00 | ± | 0.00 | ^fghij^ | 2.59 | ± | 0.10 | ^fghij^ |
| **PHE_VIN13** | 0.00 | ± | 0.00 | ^cd^ | 0.00 | ± | 0.00 | ^e^ | 2.94 | ± | 0.16 | ^ef^ |
| **PRO_BM45** | 0.00 | ± | 0.00 | ^defg^ | 0.00 | ± | 0.00 | ^fghij^ | 2.13 | ± | 0.26 | ^jklmn^ |
| **PRO_VIN13** | 0.00 | ± | 0.00 | ^defg^ | 0.00 | ± | 0.00 | ^ef^ | 2.28 | ± | 0.06 | ^ijklm^ |
| **SER_BM45** | 0.00 | ± | 0.00 | ^cdefg^ | 0.00 | ± | 0.00 | ^j^ | 3.28 | ± | 0.08 | ^de^ |
| **SER_VIN13** | 0.00 | ± | 0.000 | ^cdef^ | 0.00 | ± | 0.00 | ^hij^ | 2.85 | ± | 0.15 | ^efg^ |
| **THR_BM45** | 0.00 | ± | 0.00 | ^defg^ | 0.01 | ± | 0.00 | ^b^ | 7.78 | ± | 0.25 | ^c^ |
| **THR_VIN13** | 0.00 | ± | 0.00 | ^c^ | 0.04 | ± | 0.00 | ^a^ | 7.92 | ± | 0.59 | ^c^ |
| **TRP_BM45** | 0.00 | ± | 0.00 | ^defg^ | 0.00 | ± | 0.00 | ^fghi^ | 1.81 | ± | 0.14 | ^mnop^ |
| **TRP_VIN13** | 0.00 | ± | 0.00 | ^g^ | 0.00 | ± | 0.00 | ^efg^ | 1.49 | ± | 0.09 | ^p^ |
| **TYR_BM45** | 0.00 | ± | 0.00 | ^defg^ | 0.00 | ± | 0.00 | ^fghij^ | 2.52 | ± | 0.08 | ^fghij^ |
| **TYR_VIN13** | 0.00 | ± | 0.00 | ^cdefg^ | 0.00 | ± | 0.00 | ^ef^ | 2.77 | ± | 0.00 | ^fgh^ |
| **VAL_BM45** | 0.00 | ± | 0.00 | ^defg^ | 0.01 | ± | 0.00 | ^d^ | 1.96 | ± | 0.05 | ^klmnop^ |
| **VAL_VIN13** | 0.00 | ± | 0.00 | ^efg^ | 0.01 | ± | 0.00 | ^b^ | 1.76 | ± | 0.03 | ^nop^ |

**Table S1. continued**

|  | **Acetic Acid** | | | | **Propionic Acid** | | | | **Isobutyric Acid** | | | |
| --- | --- | --- | --- | --- | --- | --- | --- | --- | --- | --- | --- | --- |
| **ALA_BM45** | 4.17 | ± | 0.20 | ^fghijk^ | 0.02 | ± | 0.00 | ^ef^ | 0.45 | ± | 0.00 | ^c^ |
| **ALA_VIN13** | 2.22 | ± | 0.32 | ^mno^ | 0.06 | ± | 0.02 | ^bcd^ | 0.28 | ± | 0.04 | ^d^ |
| **ARG_BM45** | 6.73 | ± | 0.11 | ^bc^ | 0.01 | ± | 0.00 | ^ef^ | 0.09 | ± | 0.01 | ^fghijk^ |
| **ARG_VIN13** | 5.60 | ± | 0.07 | ^bcdef^ | 0.01 | ± | 0.00 | ^ef^ | 0.06 | ± | 0.00 | ^hijk^ |
| **ASN_BM45** | 6.76 | ± | 0.46 | ^bcde^ | 0.01 | ± | 0.00 | ^ef^ | 0.08 | ± | 0.00 | ^ghijk^ |
| **ASN_VIN13** | 4.22 | ± | 0.22 | ^efghij^ | 0.00 | ± | 0.00 | ^f^ | 0.05 | ± | 0.01 | ^ijk^ |
| **ASP_BM45** | 3.20 | ± | 0.57 | ^ijklmno^ | 0.01 | ± | 0.00 | ^ef^ | 0.14 | ± | 0.03 | ^fg^ |
| **ASP_VIN13** | 2.49 | ± | 0.96 | ^klmno^ | 0.02 | ± | 0.00 | ^ef^ | 0.10 | ± | 0.01 | ^fghij^ |
| **GLN_BM45** | 4.88 | ± | 0.83 | ^defghi^ | 0.01 | ± | 0.01 | ^ef^ | 0.07 | ± | 0.02 | ^ghijk^ |
| **GLN_VIN13** | 4.50 | ± | 0.04 | ^efghij^ | 0.01 | ± | 0.00 | ^ef^ | 0.06 | ± | 0.00 | ^hijk^ |
| **ILE_BM45** | 4.11 | ± | 0.25 | ^fghijkl^ | 0.01 | ± | 0.00 | ^ef^ | 0.22 | ± | 0.01 | ^e^ |
| **ILE_VIN13** | 4.20 | ± | 0.16 | ^efghij^ | 0.09 | ± | 0.14 | ^ef^ | 0.15 | ± | 0.05 | ^f^ |
| **LEU_BM45** | 5.08 | ± | 0.27 | ^cdefgh^ | 0.00 | ± | 0.00 | ^ef^ | 0.12 | ± | 0.01 | ^fgh^ |
| **LEU_VIN13** | 3.63 | ± | 0.88 | ^ghijklmn^ | 0.01 | ± | 0.00 | ^ef^ | 0.10 | ± | 0.01 | ^fghij^ |
| **METH_BM45** | 4.74 | ± | 0.56 | ^efghi^ | 0.04 | ± | 0.01 | ^cde^ | 0.13 | ± | 0.05 | ^fg^ |
| **METH_VIN13** | 2.29 | ± | 0.14 | ^lmno^ | 0.11 | ± | 0.00 | ^b^ | 0.07 | ± | 0.00 | ^ghijk^ |
| **NH4_BM45** | 10.72 | ± | 0.75 | ^a^ | 0.01 | ± | 0.01 | ^ef^ | 0.04 | ± | 0.00 | ^jk^ |
| **NH4_VIN13** | 6.65 | ± | 0.31 | ^bcd^ | 0.01 | ± | 0.02 | ^def^ | 0.03 | ± | 0.01 | ^k^ |
| **PHE_BM45** | 3.26 | ± | 0.29 | ^hijklmn^ | 0.01 | ± | 0.00 | ^ef^ | 0.16 | ± | 0.01 | ^ef^ |
| **PHE_VIN13** | 2.66 | ± | 0.34 | ^jklmno^ | 0.02 | ± | 0.02 | ^ef^ | 0.11 | ± | 0.03 | ^fghi^ |
| **PRO_BM45** | 4.16 | ± | 0.42 | ^fghijk^ | 0.01 | ± | 0.01 | ^ef^ | 0.15 | ± | 0.02 | ^f^ |
| **PRO_VIN13** | 1.40 | ± | 0.14 | ^o^ | 0.00 | ± | 0.00 | ^ef^ | 0.10 | ± | 0.01 | ^fghij^ |
| **SER_BM45** | 4.39 | ± | 0.33 | ^efghij^ | 0.01 | ± | 0.00 | ^ef^ | 0.44 | ± | 0.01 | ^c^ |
| **SER_VIN13** | 3.18 | ± | 0.69 | ^ijklmno^ | 0.01 | ± | 0.01 | ^ef^ | 0.43 | ± | 0.02 | ^c^ |
| **THR_BM45** | 9.22 | ± | 2.25 | ^a^ | 0.17 | ± | 0.03 | ^a^ | 0.11 | ± | 0.03 | ^fghi^ |
| **THR_VIN13** | 4.38 | ± | 0.61 | ^efghij^ | 0.16 | ± | 0.01 | ^a^ | 0.06 | ± | 0.01 | ^hijk^ |
| **TRP_BM45** | 7.04 | ± | 1.09 | ^b^ | 0.01 | ± | 0.01 | ^ef^ | 0.10 | ± | 0.01 | ^fghij^ |
| **TRP_VIN13** | 2.02 | ± | 0.07 | ^no^ | 0.00 | ± | 0.00 | ^f^ | 0.06 | ± | 0.01 | ^hijk^ |
| **TYR_BM45** | 5.18 | ± | 0.15 | ^cdefg^ | 0.01 | ± | 0.01 | ^ef^ | 0.16 | ± | 0.00 | ^ef^ |
| **TYR_VIN13** | 4.71 | ± | 0.23 | ^efghi^ | 0.03 | ± | 0.00 | ^def^ | 0.12 | ± | 0.00 | ^fgh^ |
| **VAL_BM45** | 4.02 | ± | 0.13 | ^fghijklm^ | 0.04 | ± | 0.06 | ^de^ | 1.99 | ± | 0.04 | ^a^ |
| **VAL_VIN13** | 11.70 | ± | 2.87 | ^a^ | 0.07 | ± | 0.07 | ^bc^ | 1.57 | ± | 0.18 | ^b^ |

**Table S1. continued**

|  | **Isovaleric acid** | | | | **Valeric acid*** | | | | **2-Phenylethyl acetate** | | | |
| --- | --- | --- | --- | --- | --- | --- | --- | --- | --- | --- | --- | --- |
| **ALA_BM45** | 0.04 | ± | 0.00 | ^ef^ | 0.00 | ± | 0.00 | ^b^ | 0.00 | ± | 0.00 | ^c^ |
| **ALA_VIN13** | 0.02 | ± | 0.00 | ^ef^ | 0.00 | ± | 0.00 | ^b^ | 0.00 | ± | 0.00 | ^c^ |
| **ARG_BM45** | 0.01 | ± | 0.01 | ^f^ | 0.00 | ± | 0.00 | ^b^ | 0.00 | ± | 0.00 | ^c^ |
| **ARG_VIN13** | 0.01 | ± | 0.00 | ^f^ | 0.00 | ± | 0.00 | ^b^ | 0.00 | ± | 0.00 | ^c^ |
| **ASN_BM45** | 0.01 | ± | 0.01 | ^f^ | 0.00 | ± | 0.00 | ^b^ | 0.00 | ± | 0.00 | ^c^ |
| **ASN_VIN13** | 0.02 | ± | 0.00 | ^ef^ | 0.00 | ± | 0.00 | ^b^ | 0.00 | ± | 0.00 | ^c^ |
| **ASP_BM45** | 0.01 | ± | 0.01 | ^f^ | 0.00 | ± | 0.00 | ^b^ | 0.00 | ± | 0.00 | ^c^ |
| **ASP_VIN13** | 0.00 | ± | 0.00 | ^f^ | 0.00 | ± | 0.00 | ^b^ | 0.00 | ± | 0.00 | ^c^ |
| **GLN_BM45** | 0.01 | ± | 0.01 | ^f^ | 0.00 | ± | 0.00 | ^b^ | 0.00 | ± | 0.00 | ^c^ |
| **GLN_VIN13** | 0.01 | ± | 0.00 | ^ef^ | 0.00 | ± | 0.00 | ^b^ | 0.00 | ± | 0.00 | ^c^ |
| **ILE_BM45** | 0.58 | ± | 0.02 | ^a^ | 0.00 | ± | 0.00 | ^b^ | 0.00 | ± | 0.00 | ^c^ |
| **ILE_VIN13** | 0.62 | ± | 0.03 | ^a^ | 0.00 | ± | 0.00 | ^b^ | 0.00 | ± | 0.00 | ^c^ |
| **LEU_BM45** | 0.31 | ± | 0.02 | ^d^ | 0.00 | ± | 0.00 | ^b^ | 0.00 | ± | 0.00 | ^c^ |
| **LEU_VIN13** | 0.48 | ± | 0.03 | ^b^ | 0.00 | ± | 0.00 | ^b^ | 0.00 | ± | 0.00 | ^c^ |
| **METH_BM45** | 0.02 | ± | 0.01 | ^ef^ | 0.00 | ± | 0.00 | ^b^ | 0.00 | ± | 0.00 | ^c^ |
| **METH_VIN13** | 0.01 | ± | 0.00 | ^ef^ | 0.17 | ± | 0.30 | ^a^ | 0.00 | ± | 0.00 | ^c^ |
| **NH4_BM45** | 0.00 | ± | 0.00 | ^f^ | 0.00 | ± | 0.00 | ^b^ | 0.00 | ± | 0.00 | ^c^ |
| **NH4_VIN13** | 0.00 | ± | 0.00 | ^f^ | 0.00 | ± | 0.00 | ^b^ | 0.00 | ± | 0.00 | ^c^ |
| **PHE_BM45** | 0.01 | ± | 0.01 | ^f^ | 0.00 | ± | 0.00 | ^b^ | 0.23 | ± | 0.01 | ^a^ |
| **PHE_VIN13** | 0.02 | ± | 0.02 | ^ef^ | 0.01 | ± | 0.00 | ^b^ | 0.23 | ± | 0.11 | ^b^ |
| **PRO_BM45** | 0.02 | ± | 0.00 | ^ef^ | 0.00 | ± | 0.00 | ^b^ | 0.00 | ± | 0.00 | ^c^ |
| **PRO_VIN13** | 0.03 | ± | 0.00 | ^ef^ | 0.00 | ± | 0.00 | ^b^ | 0.00 | ± | 0.00 | ^c^ |
| **SER_BM45** | 0.03 | ± | 0.03 | ^ef^ | 0.00 | ± | 0.00 | ^b^ | 0.00 | ± | 0.00 | ^c^ |
| **SER_VIN13** | 0.07 | ± | 0.01 | ^e^ | 0.00 | ± | 0.00 | ^b^ | 0.00 | ± | 0.00 | ^c^ |
| **THR_BM45** | 0.37 | ± | 0.05 | ^c^ | 0.00 | ± | 0.00 | ^b^ | 0.00 | ± | 0.00 | ^c^ |
| **THR_VIN13** | 0.39 | ± | 0.05 | ^d^ | 0.00 | ± | 0.00 | ^b^ | 0.00 | ± | 0.00 | ^c^ |
| **TRP_BM45** | 0.00 | ± | 0.00 | ^f^ | 0.00 | ± | 0.00 | ^b^ | 0.00 | ± | 0.00 | ^c^ |
| **TRP_VIN13** | 0.00 | ± | 0.00 | ^f^ | 0.00 | ± | 0.00 | ^b^ | 0.00 | ± | 0.00 | ^c^ |
| **TYR_BM45** | 0.01 | ± | 0.00 | ^ef^ | 0.00 | ± | 0.00 | ^b^ | 0.00 | ± | 0.00 | ^c^ |
| **TYR_VIN13** | 0.02 | ± | 0.01 | ^ef^ | 0.02 | ± | 0.01 | ^b^ | 0.00 | ± | 0.00 | ^c^ |
| **VAL_BM45** | 0.02 | ± | 0.00 | ^ef^ | 0.00 | ± | 0.00 | ^b^ | 0.00 | ± | 0.00 | ^c^ |
| **VAL_VIN13** | 0.02 | ± | 0.01 | ^ef^ | 0.00 | ± | 0.00 | ^b^ | 0.00 | ± | 0.00 | ^c^ |

**Table S1. continued**

|  | **2-Phenylethanol** | | | |
| --- | --- | --- | --- | --- |
| **ALA_BM45** | 0.13 | ± | 0.01 | ^c^ |
| **ALA_VIN13** | 0.13 | ± | 0.02 | ^c^ |
| **ARG_BM45** | 0.22 | ± | 0.00 | ^c^ |
| **ARG_VIN13** | 0.19 | ± | 0.00 | ^c^ |
| **ASN_BM45** | 0.48 | ± | 0.26 | ^c^ |
| **ASN_VIN13** | 0.76 | ± | 0.17 | ^c^ |
| **ASP_BM45** | 0.38 | ± | 0.01 | ^c^ |
| **ASP_VIN13** | 0.40 | ± | 0.03 | ^c^ |
| **GLN_BM45** | 0.38 | ± | 0.08 | ^c^ |
| **GLN_VIN13** | 0.31 | ± | 0.03 | ^c^ |
| **ILE_BM45** | 0.18 | ± | 0.03 | ^c^ |
| **ILE_VIN13** | 0.28 | ± | 0.24 | ^c^ |
| **LEU_BM45** | 0.24 | ± | 0.01 | ^c^ |
| **LEU_VIN13** | 0.15 | ± | 0.02 | ^c^ |
| **METH_BM45** | 0.39 | ± | 0.21 | ^c^ |
| **METH_VIN13** | 0.19 | ± | 0.01 | ^c^ |
| **NH4_BM45** | 0.18 | ± | 0.01 | ^c^ |
| **NH4_VIN13** | 0.18 | ± | 0.02 | ^c^ |
| **PHE_BM45** | 5.05 | ± | 0.08 | ^a^ |
| **PHE_VIN13** | 4.31 | ± | 1.43 | ^b^ |
| **PRO_BM45** | 0.14 | ± | 0.02 | ^c^ |
| **PRO_VIN13** | 0.19 | ± | 0.01 | ^c^ |
| **SER_BM45** | 0.29 | ± | 0.01 | ^c^ |
| **SER_VIN13** | 0.27 | ± | 0.03 | ^c^ |
| **THR_BM45** | 0.31 | ± | 0.03 | ^c^ |
| **THR_VIN13** | 0.25 | ± | 0.22 | ^c^ |
| **TRP_BM45** | 0.52 | ± | 0.02 | ^c^ |
| **TRP_VIN13** | 0.47 | ± | 0.01 | ^c^ |
| **TYR_BM45** | 0.64 | ± | 0.03 | ^c^ |
| **TYR_VIN13** | 0.44 | ± | 0.05 | ^c^ |
| **VAL_BM45** | 0.17 | ± | 0.00 | ^c^ |
| **VAL_VIN13** | 0.22 | ± | 0.02 | ^c^ |
