# Supplementary material for: The Impact of Single Amino Acids on Growth and Volatile Aroma Production by Saccharomyces cerevisiae Strains
Source: Front Microbiol. 2017 Dec 19;8:2554. doi: 10.3389/fmicb.2017.02554 (PMC5742263; doi:10.3389/fmicb.2017.02554)
Supplement: Supplementary file 2 [file Table2.docx]

Supplementary Material

**The impact of single amino acids on growth and volatile aroma production by *Saccharomyces cerevisiae* strains**

**Samantha Fairbairn^1^, Alexander McKinnon^1^, Hannibal T Musarurwa^1^, António C Ferreira^1,2^ & Florian F Bauer^1*^**

^1^Institute for Wine Biotechnology, Department of Viticulture and Oenology, University of Stellenbosch, Stellenbosch South Africa

^2^Escola Superior de Biotecnologia, Universidad Católica Portuguesa, Rua Dr. António Bernardino de Almeida, 4200-072 Porto, Portugal

*** Correspondence:**

Florian F Bauer

[fb2@sun.ac.za](mailto:fb2@sun.ac.za)

**Keywords: amino acids, nitrogen, *Saccharomyces cerevisiae*, growth kinetics, wine aroma, predictive modelling**

Table S2. The impact of increasing concentrations of leucine, isoleucine, valine, phenylalanine supplemented with either NH_4_^+^ or alanine in order to obtain a total YAN of 21.43 mM N L ^-1^. The data summarises the average fermentations and standard deviation. Additionally, the letters denote significant differences (95%) between treatments using Fisher LSD.

|  | **volatile acidity** | | | | | | | | **higher alcohols** | | | | | | | | | | | | | | | | | | |  |
| --- | --- | --- | --- | --- | --- | --- | --- | --- | --- | --- | --- | --- | --- | --- | --- | --- | --- | --- | --- | --- | --- | --- | --- | --- | --- | --- | --- | --- |
|  | **ethyl acetate** | | | | **acetic acid** | | |  | **propanol** | | |  | **isobutanol** | | |  | **butanol** | |  |  | **isoamyl alcohol** | | | | **2-phenylethanol** | | | |
| **Leu_7.14** | 0.31 | ± | 0.04 | ^a^ | 7.31 | ± | 0.38 | ^ab^ | 0.79 | ± | 0.00 | ^e^ | 0.67 | ± | 0.02 | ^d^ | 0.008 | ± | 0.000 | ^f^ | 7.50 | ± | 0.18 | ^d^ | 0.18 | ± | 0.01 | ^d^ |
| **Leu_14.28** | 0.23 | ± | 0.01 | ^bcd^ | 5.80 | ± | 0.61 | ^bcdef^ | 1.03 | ± | 0.02 | ^cd^ | 0.97 | ± | 0.16 | ^d^ | 0.008 | ± | 0.000 | ^f^ | 14.13 | ± | 0.47 | ^c^ | 0.17 | ± | 0.02 | ^d^ |
| **Leu_21.43** | 0.19 | ± | 0.01 | ^d^ | 4.39 | ± | 0.47 | ^fg^ | 1.40 | ± | 0.07 | ^a^ | 0.89 | ± | 0.10 | ^d^ | 0.008 | ± | 0.000 | ^f^ | 21.04 | ± | 2.18 | ^b^ | 0.12 | ± | 0.01 | ^d^ |
| **Ile_7.14** | 0.24 | ± | 0.01 | ^bcd^ | 7.28 | ± | 0.50 | ^abc^ | 0.54 | ± | 0.04 | ^fg^ | 0.75 | ± | 0.08 | ^d^ | 0.008 | ± | 0.000 | ^f^ | 7.85 | ± | 0.96 | ^d^ | 0.20 | ± | 0.03 | ^d^ |
| **Ile_14.28** | 0.24 | ± | 0.01 | ^bcd^ | 5.59 | ± | 0.26 | ^cdef^ | 0.70 | ± | 0.03 | ^ef^ | 0.92 | ± | 0.01 | ^d^ | 0.008 | ± | 0.000 | ^f^ | 14.65 | ± | 0.10 | ^c^ | 0.19 | ± | 0.01 | ^d^ |
| **Ile_21.43** | 0.20 | ± | 0.02 | ^d^ | 4.38 | ± | 1.12 | ^fg^ | 1.03 | ± | 0.22 | ^cd^ | 1.19 | ± | 0.36 | ^d^ | 0.008 | ± | 0.001 | ^f^ | 26.41 | ± | 6.59 | ^a^ | 0.12 | ± | 0.03 | ^d^ |
| **Val_7.14** | 0.28 | ± | 0.04 | ^ab^ | 5.87 | ± | 1.68 | ^bcdef^ | 0.86 | ± | 0.13 | ^de^ | 3.68 | ± | 1.74 | ^c^ | 0.028 | ± | 0.001 | ^a^ | 1.65 | ± | 0.06 | ^e^ | 0.19 | ± | 0.00 | ^d^ |
| **Val_14.28** | 0.24 | ± | 0.08 | ^bcd^ | 5.01 | ± | 0.77 | ^def^ | 1.16 | ± | 0.19 | ^bc^ | 9.60 | ± | 1.07 | ^b^ | 0.025 | ± | 0.002 | ^b^ | 1.53 | ± | 0.16 | ^e^ | 0.15 | ± | 0.03 | ^d^ |
| **Val_21.43** | 0.22 | ± | 0.09 | ^cd^ | 3.27 | ± | 0.65 | ^g^ | 1.30 | ± | 0.04 | ^ab^ | 14.05 | ± | 0.15 | ^a^ | 0.017 | ± | 0.001 | ^c^ | 1.37 | ± | 0.12 | ^e^ | 0.09 | ± | 0.02 | ^d^ |
| **Phe_7.14** | 0.31 | ± | 0.02 | ^a^ | 6.19 | ± | 0.46 | ^bcde^ | 0.47 | ± | 0.02 | ^g^ | 0.57 | ± | 0.03 | ^d^ | 0.011 | ± | 0.001 | ^e^ | 2.37 | ± | 0.23 | ^e^ | 7.68 | ± | 1.15 | ^c^ |
| **Phe_14.28** | 0.26 | ± | 0.03 | ^abc^ | 6.57 | ± | 0.66 | ^bcd^ | 0.50 | ± | 0.04 | ^g^ | 0.93 | ± | 0.08 | ^d^ | 0.011 | ± | 0.000 | ^e^ | 2.28 | ± | 0.04 | ^e^ | 14.28 | ± | 0.84 | ^b^ |
| **Phe_21.43** | 0.22 | ± | 0.00 | ^bcd^ | 5.58 | ± | 0.06 | ^cdef^ | 0.70 | ± | 0.02 | ^ef^ | 1.32 | ± | 0.02 | ^d^ | 0.012 | ± | 0.000 | ^de^ | 2.14 | ± | 0.05 | ^e^ | 21.21 | ± | 0.81 | ^a^ |
| **Ala_7.14** | 0.23 | ± | 0.00 | ^bcd^ | 4.88 | ± | 0.84 | ^defg^ | 1.07 | ± | 0.05 | ^c^ | 4.53 | ± | 0.69 | ^c^ | 0.011 | ± | 0.001 | ^e^ | 3.15 | ± | 0.72 | ^e^ | 0.21 | ± | 0.05 | ^d^ |
| **Ala_14.28** | 0.24 | ± | 0.01 | ^bcd^ | 4.59 | ± | 0.33 | ^efg^ | 1.10 | ± | 0.06 | ^c^ | 8.90 | ± | 1.11 | ^b^ | 0.011 | ± | 0.000 | ^e^ | 3.31 | ± | 0.44 | ^e^ | 0.21 | ± | 0.03 | ^d^ |
| **Ala_21.43** | 0.23 | ± | 0.02 | ^bcd^ | 5.46 | ± | 1.72 | ^def^ | 0.99 | ± | 0.22 | ^cd^ | 15.85 | ± | 4.30 | ^a^ | 0.012 | ± | 0.002 | ^de^ | 3.32 | ± | 0.86 | ^e^ | 0.23 | ± | 0.05 | ^d^ |
| **NH4** | 0.23 | ± | 0.02 | ^bcd^ | 8.57 | ± | 2.50 | ^a^ | 0.74 | ± | 0.15 | ^e^ | 0.85 | ± | 0.24 | ^d^ | 0.013 | ± | 0.002 | ^d^ | 2.72 | ± | 0.80 | ^e^ | 0.27 | ± | 0.08 | ^d^ |

**Table S2. continued**

|  | **acetate esters** | | | | | | | | **volatile fatty acids** | | | | | | | | | | | | | | | | | | | |
| --- | --- | --- | --- | --- | --- | --- | --- | --- | --- | --- | --- | --- | --- | --- | --- | --- | --- | --- | --- | --- | --- | --- | --- | --- | --- | --- | --- | --- |
|  | **isoamyl acetate** | | | | **2-phenylethyl acetate** | | | | **propionic acid** | | | | **isobutyric acid** | | | | **isovaleric acid** | | |  | **valeric acid** | | |  | **decanoic acid** | | |  |
| **Leu_7.14** | 0.030 | ± | 0.003 | ^c^ | 0.003 | ± | 0.000 | ^d^ | 0.04 | ± | 0.00 | ^def^ | 0.05 | ± | 0.01 | ^d^ | 0.101 | ± | 0.014 | ^d^ | 0.006 | ± | 0.001 | ^i^ | 0.001 | ± | 0.001 | ^def^ |
| **Leu_14.28** | 0.046 | ± | 0.005 | ^b^ | 0.003 | ± | 0.000 | ^d^ | 0.04 | ± | 0.00 | ^def^ | 0.06 | ± | 0.00 | ^d^ | 0.184 | ± | 0.024 | ^c^ | 0.009 | ± | 0.000 | ^efg^ | 0.001 | ± | 0.000 | ^def^ |
| **Leu_21.43** | 0.057 | ± | 0.007 | ^a^ | 0.003 | ± | 0.000 | ^d^ | 0.04 | ± | 0.00 | ^def^ | 0.09 | ± | 0.01 | ^d^ | 0.359 | ± | 0.039 | ^a^ | 0.010 | ± | 0.001 | ^de^ | 0.002 | ± | 0.000 | ^cdef^ |
| **Ile_7.14** | 0.009 | ± | 0.001 | ^ef^ | 0.003 | ± | 0.000 | ^d^ | 0.02 | ± | 0.00 | ^f^ | 0.05 | ± | 0.01 | ^d^ | 0.115 | ± | 0.014 | ^d^ | 0.007 | ± | 0.000 | ^ghi^ | 0.001 | ± | 0.000 | ^def^ |
| **Ile_14.28** | 0.018 | ± | 0.003 | ^d^ | 0.003 | ± | 0.000 | ^d^ | 0.02 | ± | 0.00 | ^f^ | 0.06 | ± | 0.01 | ^d^ | 0.222 | ± | 0.047 | ^b^ | 0.008 | ± | 0.000 | ^fgh^ | 0.001 | ± | 0.000 | ^f^ |
| **Ile_21.43** | 0.013 | ± | 0.004 | ^de^ | 0.003 | ± | 0.000 | ^d^ | 0.02 | ± | 0.00 | ^f^ | 0.08 | ± | 0.01 | ^d^ | 0.383 | ± | 0.054 | ^a^ | 0.010 | ± | 0.002 | ^ef^ | 0.002 | ± | 0.002 | ^cdef^ |
| **Val_7.14** | 0.004 | ± | 0.000 | ^gh^ | 0.003 | ± | 0.000 | ^d^ | 0.13 | ± | 0.00 | ^c^ | 0.50 | ± | 0.29 | ^cd^ | 0.023 | ± | 0.003 | ^e^ | 0.006 | ± | 0.001 | ^hi^ | 0.001 | ± | 0.000 | ^ef^ |
| **Val_14.28** | 0.003 | ± | 0.001 | ^h^ | 0.003 | ± | 0.000 | ^d^ | 0.19 | ± | 0.02 | ^b^ | 1.34 | ± | 0.15 | ^b^ | 0.021 | ± | 0.003 | ^e^ | 0.007 | ± | 0.001 | ^ghi^ | 0.001 | ± | 0.000 | ^def^ |
| **Val_21.43** | 0.003 | ± | 0.001 | ^h^ | 0.003 | ± | 0.000 | ^d^ | 0.22 | ± | 0.01 | ^a^ | 2.27 | ± | 0.06 | ^a^ | 0.023 | ± | 0.001 | ^e^ | 0.007 | ± | 0.000 | ^ghi^ | 0.000 | ± | 0.000 | ^f^ |
| **Phe_7.14** | 0.009 | ± | 0.002 | ^fg^ | 0.037 | ± | 0.010 | ^c^ | 0.03 | ± | 0.00 | ^ef^ | 0.06 | ± | 0.00 | ^d^ | 0.028 | ± | 0.002 | ^e^ | 0.012 | ± | 0.001 | ^cd^ | 0.011 | ± | 0.002 | ^a^ |
| **Phe_14.28** | 0.008 | ± | 0.001 | ^fgh^ | 0.063 | ± | 0.005 | ^b^ | 0.03 | ± | 0.00 | ^ef^ | 0.08 | ± | 0.01 | ^d^ | 0.024 | ± | 0.002 | ^e^ | 0.017 | ± | 0.001 | ^b^ | 0.010 | ± | 0.001 | ^a^ |
| **Phe_21.43** | 0.006 | ± | 0.000 | ^fgh^ | 0.085 | ± | 0.011 | ^a^ | 0.03 | ± | 0.00 | ^ef^ | 0.10 | ± | 0.01 | ^d^ | 0.021 | ± | 0.001 | ^e^ | 0.022 | ± | 0.000 | ^a^ | 0.008 | ± | 0.003 | ^b^ |
| **Ala_7.14** | 0.006 | ± | 0.001 | ^fgh^ | 0.003 | ± | 0.000 | ^d^ | 0.03 | ± | 0.00 | ^ef^ | 0.41 | ± | 0.11 | ^cd^ | 0.036 | ± | 0.008 | ^e^ | 0.013 | ± | 0.002 | ^c^ | 0.002 | ± | 0.001 | ^cde^ |
| **Ala_14.28** | 0.006 | ± | 0.001 | ^fgh^ | 0.003 | ± | 0.000 | ^d^ | 0.03 | ± | 0.00 | ^ef^ | 0.65 | ± | 0.23 | ^c^ | 0.039 | ± | 0.005 | ^e^ | 0.013 | ± | 0.001 | ^c^ | 0.003 | ± | 0.001 | ^c^ |
| **Ala_21.43** | 0.008 | ± | 0.001 | ^fgh^ | 0.003 | ± | 0.000 | ^d^ | 0.05 | ± | 0.02 | ^d^ | 1.86 | ± | 1.08 | ^a^ | 0.045 | ± | 0.014 | ^e^ | 0.013 | ± | 0.002 | ^c^ | 0.002 | ± | 0.001 | ^cd^ |
| **NH4** | 0.006 | ± | 0.002 | ^fgh^ | 0.003 | ± | 0.000 | ^d^ | 0.04 | ± | 0.01 | ^de^ | 0.08 | ± | 0.02 | ^d^ | 0.026 | ± | 0.006 | ^e^ | 0.007 | ± | 0.001 | ^ghi^ | 0.002 | ± | 0.001 | ^cd^ |
