# Supplementary material for: The Impact of Single Amino Acids on Growth and Volatile Aroma Production by Saccharomyces cerevisiae Strains
Source: Front Microbiol. 2017 Dec 19;8:2554. doi: 10.3389/fmicb.2017.02554 (PMC5742263; doi:10.3389/fmicb.2017.02554)
Supplement: Supplementary file 3 [file Table3.docx]

Supplementary Material

**The impact of single amino acids on growth and volatile aroma production by *Saccharomyces cerevisiae* strains**

**Samantha Fairbairn^1^, Alexander McKinnon^1^, Hannibal T Musarurwa^1^, António C Ferreira^1,2^ & Florian F Bauer^1*^**

^1^Institute for Wine Biotechnology, Department of Viticulture and Oenology, University of Stellenbosch, Stellenbosch South Africa

^2^Escola Superior de Biotecnologia, Universidad Católica Portuguesa, Rua Dr. António Bernardino de Almeida, 4200-072 Porto, Portugal

*** Correspondence:**

Florian F Bauer

[fb2@sun.ac.za](mailto:fb2@sun.ac.za)

**Keywords: amino acids, nitrogen, *Saccharomyces cerevisiae*, growth kinetics, wine aroma, predictive modelling**

# Table S3. The impact of changing the concentration of individual branched chain and aromatic amino acids in complex amino acid mixtures on the production of volatile compounds by VIN13, as determined by GC-FID. Fermentations contained 14.3 mmol N L^-1^ of YAN, of which 3.57 mmol N  L^- 1^ was provided by ammonium chloride, the remainder was made up of all the amino acids, where each one provided equal amounts of fermentable nitrogen except for leucine, isoleucine, valine, phenylalanine, tyrosine, and threonine which were also either absent (0) or present at twice (2) the concentration of the other amino acids. The data summarizes the average fermentations (mg/L) and standard deviation. Additionally, the letters denote significant differences (95%) between treatments using Fisher LSD.

|  | **All Amino acids** | | |  | **NH4** |  |  |  | **0 Isoleucine** | | |  | **2 Isoleucine** | | |  | **0 Leucine** | |  |  | **2 Leucine** | |  |  |
| --- | --- | --- | --- | --- | --- | --- | --- | --- | --- | --- | --- | --- | --- | --- | --- | --- | --- | --- | --- | --- | --- | --- | --- | --- |
| **2-phenyl ethanol** | 53.78 | ± | 3.78 | ^cd^ | 10.24 | ± | 0.69 | ^a^ | 53.64 | ± | 2.41 | ^cd^ | 50.40 | ± | 3.97 | ^c^ | 62.52 | ± | 0.51 | ^f^ | 57.50 | ± | 2.68 | ^de^ |
| **2-phenylethyl acetate** | 1.14 | ± | 0.06 | ^bc^ | 0.42 | ± | 0.00 | ^a^ | 1.25 | ± | 0.07 | ^bcd^ | 1.07 | ± | 0.12 | ^b^ | 1.36 | ± | 0.02 | ^d^ | 1.27 | ± | 0.08 | ^cd^ |
| **3-ethoxy-1-propanol** | 2.59 | ± | 0.22 | ^de^ | 3.43 | ± | 0.38 | ^f^ | 2.63 | ± | 0.16 | ^de^ | 2.29 | ± | 0.20 | ^c^ | 2.83 | ± | 0.03 | ^e^ | 2.18 | ± | 0.09 | ^bc^ |
| **acetic acid** | 575.27 | ± | 19.11 | ^bc^ | 656.16 | ± | 4.67 | ^ef^ | 609.67 | ± | 7.46 | ^cd^ | 590.48 | ± | 18.83 | ^bcd^ | 671.17 | ± | 8.13 | ^f^ | 623.85 | ± | 22.52 | ^de^ |
| **butanol** | 0.57 | ± | 0.02 | ^bc^ | 0.70 | ± | 0.04 | ^f^ | 0.60 | ± | 0.01 | ^de^ | 0.56 | ± | 0.01 | ^bc^ | 0.61 | ± | 0.02 | ^e^ | 0.50 | ± | 0.01 | ^a^ |
| **butyric acid** | 1.05 | ± | 0.05 | ^ab^ | 1.00 | ± | 0.01 | ^a^ | 1.06 | ± | 0.03 | ^abc^ | 1.04 | ± | 0.04 | ^ab^ | 1.04 | ± | 0.07 | ^ab^ | 1.13 | ± | 0.03 | ^cd^ |
| **ethyl acetate** | 45.74 | ± | 3.35 | ^cde^ | 48.26 | ± | 3.28 | ^de^ | 36.57 | ± | 0.43 | ^a^ | 32.80 | ± | 3.35 | ^a^ | 45.74 | ± | 1.49 | ^cde^ | 41.62 | ± | 2.93 | ^bc^ |
| **ethyl caprate** | 0.15 | ± | 0.07 | ^d^ | 0.07 | ± | 0.01 | ^a^ | 0.06 | ± | 0.00 | ^a^ | 0.08 | ± | 0.04 | ^ab^ | 0.13 | ± | 0.02 | ^bcd^ | 0.22 | ± | 0.02 | ^e^ |
| **ethyl caprylate** | 0.56 | ± | 0.29 | ^bcd^ | 0.32 | ± | 0.05 | ^a^ | 0.32 | ± | 0.04 | ^a^ | 0.37 | ± | 0.11 | ^ab^ | 0.60 | ± | 0.05 | ^cde^ | 1.03 | ± | 0.18 | ^g^ |
| **hexanoic acid** | 3.02 | ± | 0.16 | ^bcde^ | 2.71 | ± | 0.05 | ^ab^ | 3.18 | ± | 0.13 | ^cde^ | 2.90 | ± | 0.28 | ^abcd^ | 2.63 | ± | 0.27 | ^a^ | 3.34 | ± | 0.16 | ^e^ |
| **isoamyl acetate** | 1.23 | ± | 0.30 | ^abc^ | 1.23 | ± | 0.08 | ^abc^ | 1.02 | ± | 0.32 | ^a^ | 1.10 | ± | 0.29 | ^a^ | 1.73 | ± | 0.07 | ^ef^ | 2.21 | ± | 0.02 | ^g^ |
| **isobutanol** | 25.58 | ± | 2.73 | ^d^ | 13.21 | ± | 0.36 | ^a^ | 21.89 | ± | 2.80 | ^bc^ | 25.45 | ± | 3.52 | ^d^ | 25.47 | ± | 0.75 | ^d^ | 25.98 | ± | 0.72 | ^d^ |
| **isobutyric acid** | 1.78 | ± | 0.15 | ^cd^ | 0.87 | ± | 0.01 | ^a^ | 1.53 | ± | 0.20 | ^b^ | 1.80 | ± | 0.20 | ^cde^ | 1.64 | ± | 0.10 | ^bc^ | 1.94 | ± | 0.05 | ^def^ |
| **propanol** | 25.60 | ± | 2.48 | ^f^ | 48.17 | ± | 4.04 | ^g^ | 25.41 | ± | 0.51 | ^ef^ | 21.91 | ± | 1.44 | ^cd^ | 23.25 | ± | 0.60 | ^cdef^ | 22.44 | ± | 1.23 | ^cde^ |
| **propionic acid** | 1.29 | ± | 0.04 | ^cd^ | 1.97 | ± | 0.04 | ^g^ | 1.57 | ± | 0.03 | ^f^ | 1.17 | ± | 0.02 | ^a^ | 1.23 | ± | 0.04 | ^abc^ | 1.40 | ± | 0.06 | ^e^ |
| **valeric acid** | 0.53 | ± | 0.02 | ^abc^ | 0.51 | ± | 0.03 | ^abc^ | 0.56 | ± | 0.11 | ^bcd^ | 0.45 | ± | 0.10 | ^ab^ | 0.61 | ± | 0.02 | ^cd^ | 0.48 | ± | 0.06 | ^ab^ |

**Table S3. continued**

|  | **0 Valine** | |  |  | **2 Valine** | |  |  | **0 Tryptophan** | | |  | **2 Tryptophan** | | |  | **0 Tyrosine** | | |  | **2 Tyrosine** | | |  |
| --- | --- | --- | --- | --- | --- | --- | --- | --- | --- | --- | --- | --- | --- | --- | --- | --- | --- | --- | --- | --- | --- | --- | --- | --- |
| **2-phenyl ethanol** | 67.89 | ± | 5.28 | ^g^ | 55.68 | ± | 1.26 | ^de^ | 50.35 | ± | 1.37 | ^c^ | 55.35 | ± | 2.39 | ^d^ | 59.77 | ± | 1.42 | ^ef^ | 55.46 | ± | 2.24 | ^de^ |
| **2-phenylethyl acetate** | 1.35 | ± | 0.11 | ^d^ | 1.29 | ± | 0.09 | ^cd^ | 1.10 | ± | 0.03 | ^bc^ | 1.22 | ± | 0.08 | ^bcd^ | 1.37 | ± | 0.15 | ^d^ | 1.24 | ± | 0.08 | ^bcd^ |
| **3-ethoxy-1-propanol** | 2.76 | ± | 0.14 | ^e^ | 2.34 | ± | 0.10 | ^cd^ | 2.28 | ± | 0.06 | ^c^ | 2.34 | ± | 0.22 | ^cd^ | 1.80 | ± | 0.03 | ^a^ | 2.46 | ± | 0.22 | ^cd^ |
| **acetic acid** | 625.01 | ± | 26.08 | ^de^ | 564.03 | ± | 34.43 | ^ab^ | 656.47 | ± | 22.35 | ^ef^ | 603.27 | ± | 19.33 | ^cd^ | 584.12 | ± | 26.84 | ^bc^ | 560.63 | ± | 35.40 | ^ab^ |
| **butanol** | 0.54 | ± | 0.02 | ^b^ | 0.62 | ± | 0.02 | ^e^ | 0.54 | ± | 0.02 | ^b^ | 0.55 | ± | 0.01 | ^bc^ | 0.54 | ± | 0.02 | ^b^ | 0.58 | ± | 0.01 | ^cd^ |
| **butyric acid** | 1.15 | ± | 0.08 | ^d^ | 1.11 | ± | 0.05 | ^bcd^ | 1.13 | ± | 0.02 | ^cd^ | 1.07 | ± | 0.03 | ^abcd^ | 1.11 | ± | 0.08 | ^bcd^ | 1.07 | ± | 0.04 | ^abc^ |
| **ethyl acetate** | 44.10 | ± | 3.57 | ^bcd^ | 36.50 | ± | 0.26 | ^a^ | 49.22 | ± | 1.32 | ^e^ | 41.99 | ± | 3.36 | ^bc^ | 34.32 | ± | 2.51 | ^a^ | 41.22 | ± | 0.79 | ^b^ |
| **ethyl caprate** | 0.14 | ± | 0.03 | ^cd^ | 0.13 | ± | 0.02 | ^bcd^ | 0.09 | ± | 0.01 | ^abc^ | 0.09 | ± | 0.02 | ^abcd^ | 0.14 | ± | 0.06 | ^cd^ | 0.11 | ± | 0.06 | ^abcd^ |
| **ethyl caprylate** | 0.73 | ± | 0.12 | ^de^ | 0.79 | ± | 0.13 | ^ef^ | 0.70 | ± | 0.04 | ^cde^ | 0.73 | ± | 0.04 | ^de^ | 0.97 | ± | 0.06 | ^fg^ | 0.51 | ± | 0.16 | ^abc^ |
| **hexanoic acid** | 3.27 | ± | 0.35 | ^de^ | 3.20 | ± | 0.12 | ^cde^ | 3.07 | ± | 0.11 | ^bcde^ | 3.12 | ± | 0.15 | ^cde^ | 3.01 | ± | 0.27 | ^bcde^ | 3.09 | ± | 0.22 | ^cde^ |
| **isoamyl acetate** | 1.70 | ± | 0.04 | ^ef^ | 1.41 | ± | 0.11 | ^bcd^ | 1.69 | ± | 0.11 | ^ef^ | 1.56 | ± | 0.16 | ^def^ | 1.16 | ± | 0.05 | ^ab^ | 1.46 | ± | 0.08 | ^cde^ |
| **isobutanol** | 19.32 | ± | 1.62 | ^b^ | 46.04 | ± | 1.95 | ^g^ | 33.69 | ± | 1.90 | ^f^ | 24.50 | ± | 1.20 | ^cd^ | 26.92 | ± | 1.51 | ^de^ | 26.54 | ± | 2.78 | ^de^ |
| **isobutyric acid** | 1.55 | ± | 0.12 | ^b^ | 3.21 | ± | 0.18 | ^g^ | 1.98 | ± | 0.08 | ^def^ | 1.97 | ± | 0.14 | ^def^ | 1.90 | ± | 0.05 | ^def^ | 2.00 | ± | 0.07 | ^ef^ |
| **propanol** | 21.21 | ± | 1.24 | ^bcd^ | 23.41 | ± | 2.61 | ^def^ | 25.61 | ± | 1.00 | ^f^ | 20.33 | ± | 0.91 | ^abc^ | 18.67 | ± | 0.87 | ^ab^ | 22.92 | ± | 1.68 | ^cdef^ |
| **propionic acid** | 1.28 | ± | 0.05 | ^cd^ | 1.42 | ± | 0.08 | ^e^ | 1.30 | ± | 0.01 | ^d^ | 1.23 | ± | 0.03 | ^abc^ | 1.24 | ± | 0.02 | ^bc^ | 1.29 | ± | 0.02 | ^cd^ |
| **valeric acid** | 0.67 | ± | 0.11 | ^d^ | 0.56 | ± | 0.06 | ^bcd^ | 0.45 | ± | 0.04 | ^ab^ | 0.52 | ± | 0.12 | ^abc^ | 0.42 | ± | 0.12 | ^a^ | 0.54 | ± | 0.11 | ^abcd^ |

**Table S3. continued**

|  | **0 Phenylalanine** | | |  | **2 Phenylalanine** | | |  |
| --- | --- | --- | --- | --- | --- | --- | --- | --- |
| **2-phenyl ethanol** | 23.45 | ± | 0.77 | ^b^ | 122.33 | ± | 2.99 | ^h^ |
| **2-phenylethyl acetate** | 0.61 | ± | 0.00 | ^a^ | 2.40 | ± | 0.34 | ^e^ |
| **3-ethoxy-1-propanol** | 1.93 | ± | 0.13 | ^ab^ | 1.95 | ± | 0.13 | ^ab^ |
| **acetic acid** | 563.43 | ± | 16.56 | ^ab^ | 537.57 | ± | 11.80 | ^a^ |
| **butanol** | 0.56 | ± | 0.00 | ^bc^ | 0.58 | ± | 0.03 | ^cd^ |
| **butyric acid** | 1.10 | ± | 0.02 | ^bcd^ | 1.09 | ± | 0.04 | ^bcd^ |
| **ethyl acetate** | 43.87 | ± | 0.95 | ^bc^ | 41.92 | ± | 3.54 | ^bc^ |
| **ethyl caprate** | 0.08 | ± | 0.00 | ^ab^ | 0.07 | ± | 0.01 | ^a^ |
| **ethyl caprylate** | 0.62 | ± | 0.08 | ^cde^ | 0.51 | ± | 0.12 | ^abc^ |
| **hexanoic acid** | 2.89 | ± | 0.12 | ^abc^ | 2.93 | ± | 0.43 | ^abcd^ |
| **isoamyl acetate** | 1.74 | ± | 0.01 | ^f^ | 1.55 | ± | 0.17 | ^def^ |
| **isobutanol** | 29.64 | ± | 0.88 | ^e^ | 26.92 | ± | 2.05 | ^de^ |
| **isobutyric acid** | 2.10 | ± | 0.13 | ^f^ | 1.83 | ± | 0.14 | ^cde^ |
| **propanol** | 17.45 | ± | 0.73 | ^a^ | 18.86 | ± | 1.98 | ^ab^ |
| **propionic acid** | 1.21 | ± | 0.01 | ^ab^ | 1.20 | ± | 0.02 | ^ab^ |
| **valeric acid** | 0.54 | ± | 0.02 | ^abcd^ | 0.53 | ± | 0.04 | ^abc^ |
